# *In Vivo* Genome and Methylome Adaptation of *cag*-Negative Helicobacter pylori during Experimental Human Infection

**DOI:** 10.1128/mBio.01803-20

**Published:** 2020-08-25

**Authors:** Iratxe Estibariz, Florent Ailloud, Sabrina Woltemate, Boyke Bunk, Cathrin Spröer, Jörg Overmann, Toni Aebischer, Thomas F. Meyer, Christine Josenhans, Sebastian Suerbaum

**Affiliations:** a Medical Microbiology and Hospital Epidemiology, Max von Pettenkofer Institute, Faculty of Medicine, LMU Munich, Munich, Germany; b Institute of Medical Microbiology and Hospital Epidemiology, Hannover Medical School, Hannover, Germany; c German Center for Infection Research (DZIF), Munich and Hannover-Braunschweig Sites, Germany; d Leibniz Institute DSMZ-German Collection of Microorganisms and Cell Cultures, Braunschweig, Germany; e Department of Molecular Biology, Max Planck Institute for Infection Biology, Berlin, Germany; GSK Vaccines

**Keywords:** DNA methylation, *Helicobacter pylori*, adaptive mutations, genome analysis, SMRT sequencing, PacBio, MiSeq Illumina, DNA modification, methylome

## Abstract

Multiple studies have demonstrated rapid bacterial genome evolution during chronic infection with Helicobacter pylori. In contrast, little was known about genetic changes during the first stages of infection, when selective pressure is likely to be highest. Using single-molecule, real-time (SMRT) and Illumina sequencing technologies, we analyzed genome and methylome evolution during the first 10 weeks of infection by comparing the *cag* pathogenicity island (*cag*PAI)-negative H. pylori challenge strain BCS 100 with pairs of H. pylori reisolates from gastric antrum and corpus biopsy specimens of 10 human volunteers who had been infected with this strain as part of a vaccine trial. Most genetic changes detected in the reisolates affected genes with a surface-related role or a predicted function in peptide uptake. Apart from phenotypic changes of the bacterial envelope, a duplication of the catalase gene was observed in one reisolate, which resulted in higher catalase activity and improved survival under oxidative stress conditions. The methylomes also varied in some of the reisolates, mostly by activity switching of phase-variable methyltransferase (MTase) genes. The observed *in vivo* mutation spectrum was remarkable for a very high proportion of nonsynonymous mutations. Although the data showed substantial within-strain genome diversity in the challenge strain, most antrum and corpus reisolates from the same volunteers were highly similar to each other, indicating that the challenge infection represents a major selective bottleneck shaping the transmitted population. Our findings suggest rapid *in vivo s*election of H. pylori during early-phase infection providing adaptation to different individuals by common mechanisms of genetic and epigenetic alterations.

## Abstract (precis)

Exceptional genetic diversity and variability are hallmarks of Helicobacter pylori, but the biological role of this plasticity remains incompletely understood. Here, we had the rare opportunity to investigate the molecular evolution during the first weeks of H. pylori infection by comparing the genomes and epigenomes of H. pylori strain BCS 100 used to challenge human volunteers in a vaccine trial with those of bacteria reisolated from the volunteers 10 weeks after the challenge. The data provide molecular insights into the process of establishment of this highly versatile pathogen in 10 different human individual hosts, showing, for example, selection for changes in host-interaction molecules as well as changes in epigenetic methylation patterns. The data provide important clues to the early adaptation of H. pylori to new host niches after transmission, which we believe is vital to understand its success as a chronic pathogen and develop more efficient treatments and vaccines.

## INTRODUCTION

Helicobacter pylori is a highly prevalent human pathogen that causes chronic inflammation of the stomach epithelium. Although the majority of infected individuals do not develop further symptoms, H. pylori infection can give rise to clinical disease, including gastroduodenal ulcers, lymphoma of the mucosa-associated lymphoid tissue (MALT), and gastric cancer (1, 2). H. pylori has been recognized as a class I carcinogenic agent by the World Health Organization (WHO) since 1994 (3). If not treated, this human gastric pathogen usually establishes a lifelong infection (2, 4). Transmission mostly takes place during childhood due to direct contact with infected relatives, but the infection is typically diagnosed during adult life (2, 5).

A successful colonization of the stomach niche requires, among many other traits, urease activity (6, 7), the ability of H. pylori to swim in the mucus layer using flagellum-based chemotactic motility (8, 9), and multiple adhesins to attach to the gastric epithelium (10, 11).

Genome studies of sequential H. pylori isolates from chronically infected individuals demonstrated that H. pylori undergoes rapid *in vivo* genome evolution (12–14). This is in part a result of the natural competence of this bacterium, which allows it to take up extracellular DNA, leading to chromosome exchange during mixed infection with other H. pylori strains meeting in the same stomach niche. Moreover, a high mutation rate contributes to an elevated genetic diversity (15). The high mutation rate results from the lack of genes involved in a traditional mismatch repair (MMR) system (16, 17) and mutagenic properties of the H. pylori polymerase I (Pol I) enzyme (18). Genes with an outer membrane-related role vary at an elevated apparent rate, probably due to positive selection (14, 19, 20), reinforcing the idea that genes involved in adhesion and host interaction play a vital role in establishing and maintaining the chronic infection.

Furthermore, H. pylori has a relatively small genome but encodes a large number of restriction-modification (R-M) systems. Every H. pylori strain carries a unique set of R-M systems, leading to variable methylomes (21, 22). It was recently verified that R-M systems act as barriers against heterologous incoming DNA (e.g., antibiotic resistance cassettes), while restriction endonucleases (REases) do not limit the import of homeologous DNA from other H. pylori strains (23). In addition to the role of methyltransferases (MTases) in self-DNA protection as parts of R-M systems, methylation controls several bacterial functions by regulating the expression of genes. Many MTase genes include repetitive nucleotide tracts prone to phase variation by slipped strand mispairing. It has been suggested that phase-variable MTases might play a role in the adaptation of H. pylori and other pathogens to new hosts due to the regulation of the expression of genes involved in colonization and pathogenesis (24–26).

So far, little is known about the genome and especially the methylome evolution of H. pylori during the early phases of the infection and the role of these changes in the pathogen’s adaptation to different stomach conditions. How H. pylori genomic diversity influences the ability of the bacteria to colonize and survive in a new stomach niche is not well understood. To our knowledge, only one previous study from our group has explored whole-genome and methylome adaptation to new stomach niches during an experimental human infection. In that prior study (20), the H. pylori strain BCM-300 was used to challenge human volunteers as part of a vaccine trial (27). BCM-300 carries a functional *cag* pathogenicity island (*cag*PAI), generating higher levels of inflammation. However, only one H. pylori reisolate per individual was available from that study, so that we could not study heterogeneity of the H. pylori population within one stomach during this early-stage infection.

In the present study, we used single-molecule, real-time (SMRT) sequencing technology to obtain and compare finished closed genome sequences and methylomes of the challenge strain and pairs of H. pylori reisolates from the antrum and corpus of 10 volunteers who participated in an experimental infection study performed with the *cag*PAI-negative challenge strain BCS 100 (28, 29), with the aim of detecting genomic and epigenomic alterations during early infection. In addition, we compared the genome sequences of 16 purified single colonies from the parental (challenge) strain to assess the homogeneity of the inoculum. Whole-genome and methylome comparisons revealed substantial genomic diversification during early human infection affecting outer membrane-related genes and, interestingly, genes involved in peptide uptake. In addition to nucleotide sequence changes, switching of the activity of phase-variable MTases generated methylome variability between reisolates, pointing to a role of methylation in adaptation via altering the expression of genes.

## RESULTS

### Genome analysis of the BCS 100 challenge strain.

The *cag*PAI-negative H. pylori strain BCS 100 was originally isolated from a patient with mild superficial gastritis and was the first H. pylori strain used for a challenge study in human volunteers (29). In contrast to most H. pylori strains used for research, BCS 100 had not been subjected to purification from single colonies, in order to preserve its full infectivity. Subclones H1 to H16 were subsequently purified from BCS 100 single colonies, and a draft genome sequence of the H1 clone had already been obtained previously, by 454 sequencing (19). In order to be able to understand the dynamics of genome and methylome adaptation during early-stage human infection with BCS 100, it was important to initially assess the within-population diversity of the challenge strain. We used SMRT sequencing technology to obtain the complete genome sequence and the methylome of one single colony-purified clone (H1) of BCS 100. In addition, we used Illumina MiSeq technology to obtain draft genomes from the 15 other clones (H2 to H16). In total, we analyzed 16 genomes from the BCS 100 input strain population. Among clones H1 to H16, single nucleotide polymorphisms (SNPs) were identified at 16 positions (see Table S3A in the supplemental material). Individual clones H2 to H16 differed from the reference clone H1 at one to five positions each, and none of the clones H2 to H16 was identical to H1 at all 16 polymorphic positions. Eighty percent of the polymorphic positions were nonsynonymous. In addition to the single nucleotide polymorphisms, the comparisons between H1 and H2 to H16 identified four clusters of nucleotide polymorphisms (CNPs; see Materials and Methods for definition) in four different clones (Table S3B). The four CNPs and five out of the 16 SNPs were located within a specificity subunit of a predicted type I R-M system with yet-unknown target motif and activity. The MTase of this R-M system displays more than 90% nucleotide identity with M.HpyAXIII from the H. pylori strain 26695 (30). The type I R-M system consists of one MTase gene, one REase gene, and two S subunits. The two S subunits are almost identical in sequence and located in two different genomic locations. This situation could be the result either of a recent duplication or of concerted evolution (31). All the polymorphisms found in the study affected only the second S subunit in both loci which in one locus was located between the first S subunit and the REase of the type I R-M system (position in H1: 841163 to 841921) and in the other location between the outer membrane protein (OMP) gene *hofF* and the duplicated S subunit (position in H1: 786359 to 787114). The four CNPs did not share any polymorphisms. This overall small number of polymorphisms indicated a low level of heterogeneity within the challenge strain population, consistent with known patterns of within-host strain diversity (32). No evidence of an ongoing mixed infection with unrelated H. pylori strains was obtained.

### Whole-genome comparison of the challenge strain and reisolates.

**FIG 1:**
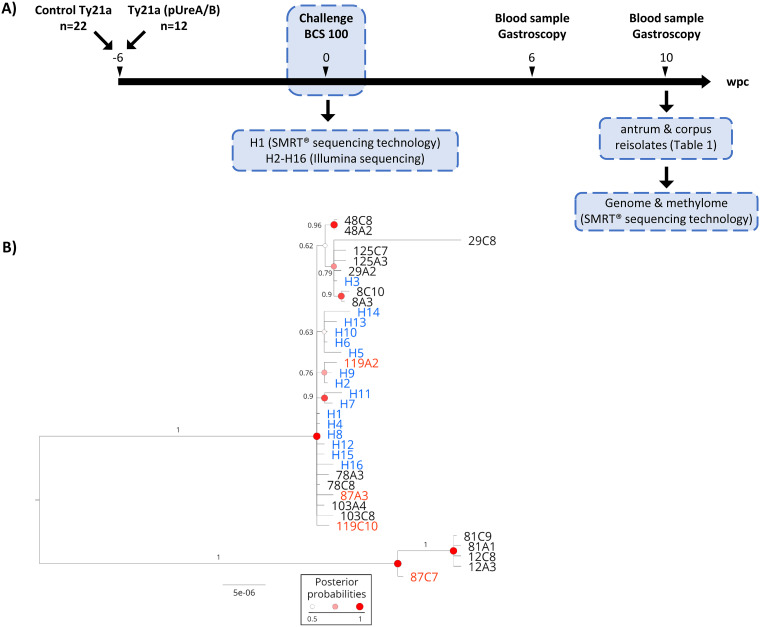
(A) Schematic representation of the vaccine/challenge trial (28) which was the basis of the present study. Human volunteers were given a *Salmonella* Ty21a live vaccine control or a recombinant Ty21a vaccine expressing the H. pylori urease. Forty-two days later, human volunteers were challenged with the *cag*PAI-negative BCS 100 H. pylori strain, a heterogeneous strain from which several subclones (H1 to H16) were genome sequenced. Six and 10 weeks postchallenge (wpc), gastroscopies were performed and blood samples were collected. Reisolates were cultured from antrum and corpus biopsy specimens taken 10 wpc, and reisolates from 10 volunteers were subjected to SMRT sequencing technology to obtain complete genome sequences and methylome data. (B) Phylogenetic tree representing the genomes of all strains. The reisolates 12A3, 12C8, 81A1, 81C9, and 87C7 represent a distinct subpopulation compared to the clones obtained from the challenge strain H1 to H16. Blue, H1 to H16 clones. Red, volunteers infected by reisolates descending from distinct subpopulations of the inoculum. The scale bar indicates substitutions per site. Statistical branch support is indicated by posterior probability values and node circles.

In order to assess genome and methylome changes during early-stage infection of human volunteers with H. pylori BCS 100, pairs of H. pylori reisolates were recovered from antrum and corpus biopsy specimens obtained from the 10 human volunteers from the challenge group of the vaccine study (five each randomly selected from the vaccine and control arms of the study), at 10 weeks after H. pylori challenge (28). For all 20 reisolates, finished closed genome sequences including methylome data were obtained using SMRT sequencing technology (Fig. 1A).

We first studied how the intrinsic diversity observed in clones H1 to H16 of BCS 100 was reflected in the reisolates. Eleven of the 20 reisolates were identical to H1 at all 16 SNP positions. Most (13/16) of the single polymorphic nucleotides identified among clones H1 to H16 were not polymorphic among the reisolates, which all were identical to H1 at these positions. Only three of the polymorphisms detected among the inoculum clones were also detected in nine reisolates, in different combinations. Notably, a combination of two nonsynonymous SNPs in clone H3, affecting a predicted multidrug efflux transporter and the *pdxJ* gene, was also found in six reisolates (8A3, 8C10, 29A2, 29C8, 125A2, and 125C7), suggesting that those reisolates and clone H3 derive from a recent common ancestor. A nonsynonymous SNP in the *copA* gene, found in H2 and H9 of the input population, was also present in reisolate 119A2 (Table S3A).

Since sequencing 16 clones from the inoculum may not fully capture the inherent population variability, we next analyzed genetic variants that were present in the reisolates but not detected in any of clones H1 to H16. We observed 86 SNPs shared among reisolates from different volunteers, here called non-unique SNPs. Eighty-three non-unique SNPs were present in the same group of reisolates (12A3, 12C8, 81A1, 81C9, and 87C7), and three were present in the same group of reisolates except 87C7. In addition, we detected six CNPs shared between these five reisolates and one CNP shared between 12A3, 12C8, 81A1, and 81C9, many of which were within the outer membrane protein (OMP)-encoding genes *babA*, *babB*, *hopQ*, and *hofC.* One CNP was located within the S subunit of a putative type I R-M system with unknown target sequence and activity status, and one CNP was located in an intergenic region (Table S3C). The data are most compatible with an *in vivo* selection of a subpopulation that was likely present in the heterogeneous input strain population BCS 100 but not represented by clones H1 to H16. Reisolates 12A3, 12C8, 81A1, 81C9, and 87C7 might have all evolved from this precursor. In agreement with this, these reisolates all cluster together in a phylogenetic tree (Fig. 1B). We note that most antrum and corpus reisolates from the same volunteer were very closely related and more similar to each other than to any other strain in the tree (Fig. 1B). Only a few reisolate clones, including paired antrum and corpus clones from two volunteers, clustered apart from each other and from the inoculum diversity sampled in H1 to H16 (Fig. 1B). The common clustering of reisolates from most volunteers suggests a colonization by very similar lineages selected from the heterogeneous inoculum.

We next investigated unique polymorphisms present in either one single reisolate (unique SNPs) or the two paired reisolates collected from the same volunteer (pair-specific SNPs). Those genetic modifications are most likely to have emerged *de novo*, during the short duration of the challenge infection (10 weeks). Whole-genome analysis of all 20 reisolates versus the 16 colonies from the challenge strain revealed a total of 25 SNPs that comprised 23 unique SNPs, and 2 pair-specific SNPs (Table 1). We also detected one unique CNP in one reisolate (Table S3C). The number of unique SNPs per reisolate varied between 0 and 3. The average mutation rate was 4.5 × 10^−6^ mutations per site per year. The mutation rates for the antrum and corpus reisolates were 5.0 × 10^−6^ and 4.0 × 10^−6^ mutations per site per year, respectively. The two pair-specific SNPs were excluded from this analysis, since they were present in both antrum and corpus reisolates from one infected volunteer, and they are therefore likely to already have been present in the inoculum, despite not being represented in clones H1 to H16. The mutation rates for control and vaccination groups were 5.0 × 10^−6^ and 4.0 × 10^−6^ mutations per site per year (Table 1), respectively. Statistical analysis of the mutation frequencies (unpaired *t* test) did not show a significant difference between the mutation rates (antrum versus corpus, *P* = 0.4566, and vaccinated versus control, *P* = 0.4585). Taken together, the mutation rates calculated here were in agreement with previous studies (13, 19, 20). A very large proportion of unique SNPs (76%) were nonsynonymous, probably resulting from a combination of diversifying selection and a lack of time to purge slightly deleterious nonsynonymous mutations (see Discussion). Most unique SNPs were detected in genes belonging to the functional category of “Cell envelope, transport and binding proteins” (genolist.pasteur.fr/PyloriGene/) (Table 2). In the subsequent paragraphs, we will highlight a selection of observed changes.

**TABLE 1:** Key characteristics of the genome sequences of H. pylori reisolates from human volunteers challenged with strain BCS 100^*a*^

Strain	Location	Genome length (bp)	No. of SNPs			Mutation rate changes/site/yr
			Unique	Pair specific	Total	
H1	Challenge	1,563,305				
8A3	Antrum	1,563,267	1	1	2	6.67E−06
8C10	Corpus	1,563,260	1	1	2	6.67E−06
12A3	Antrum	1,563,420	1	0	1	3.34E−06
12C8	Corpus	1,563,409	1	0	1	3.34E−06
29A2	Antrum	1,563,333	1	0	1	3.34E−06
29C8	Corpus	1,561,020	2	0	2	6.68E−06
48A2	Antrum	1,563,280	0	1	1	3.34E−06
48C8	Corpus	1,563,290	0	1	1	3.34E−06
78A3	Antrum	1,563,274	2	0	2	6.67E−06
78C8	Corpus	1,563,292	0	0	0	0.00E+00
81A1	Antrum	1,563,405	1	0	1	3.34E−06
81C9	Corpus	1,563,438	0	0	0	0.00E+00
87A3	Antrum	1,563,302	3	0	3	1.00E−05
87C7	Corpus	1,563,411	0	0	0	0.00E+00
103A4	Antrum	1,563,266	1	0	1	3.34E−06
103C8	Corpus	1,566,721	3	0	3	9.98E−06
125A2	Antrum	1,563,276	1	0	1	3.34E−06
125C7	Corpus	1,563,239	2	0	2	6.67E−06
119A2	Antrum	1,563,232	2	0	2	6.67E−06
119C10	Corpus	1,563,317	1	0	1	3.34E−06
Total			23	2	25	4.50E−06

a The letters A and C in the reisolate names indicates whether a strain was cultured from antrum (A) or corpus (C) biopsy specimens.

**TABLE 2:** Unique and pair-specific SNPs in *H. pylori* reisolates from infected volunteers^*a*^

Functional category and no.	Strain(s)	Position	From > to	Type	ORF	HP no.
Energy metabolism						
1	78A3	317530	c > t	Nonsynonymous	*pgi*	HP1166
2	87A3	622512	c > t	Nonsynonymous	*hydA*	HP0631
3	8A3, 8C10	1407887	c > t	Nonsynonymous	*putA*	HP0056
Cell envelope, transport, and binding proteins						
4	12C8	33480	a > g	Nonsynonymous	*copA*	HP1503
5	81A1	34327	t > c	Nonsynonymous	*copA*	HP1503
6	29A2	231005	g > a	Nonsynonymous	*babA*	HP1243
7	8C10	231370	c > a	Stop	*babA*	HP1243
8	8A3	232590	g > a	Nonsynonymous	*babA*	HP1243
9	87A3	896139	c > a	Nonsynonymous	*oppB*	HP1251
10	119C10	1185884	g > a	Nonsynonymous	*rfaC*	HP0279
11	119A2	1211998	g > a	Nonsynonymous	*oppC*	HP0251
12	125A3	1213476	g > a	Nonsynonymous	*oppD*	HP0250
13	78A3	1213737	g > t	Stop	*oppD*	HP0250
14	87A3	1213789	g > a	Nonsynonymous	*oppD*	HP0250
DNA metabolism						
15	12A3	815510	t > c	Synonymous	*uvrC*	HP0821
Protein synthesis						
16	29C8	1336072	c > t	Nonsynonymous	*thrS*	HP0123
Regulatory functions						
17	103A4	1298936	c > t	Nonsynonymous	*arcS*	HP0164
Unknown and hypothetical						
18	125C7	166082	c > t	Nonsynonymous	NA	HP0953
19	29C8	332008	a > g	Nonsynonymous	NA	HP1154
20	103C8	426225	c > t	Nonsynonymous	NA	HP0394
21	103C8	1303279	c > t	Nonsynonymous	*hcpD*	HP0160
22	48A2, 48C8	1331716	c > t	Nonsynonymous	NA	HP0130
23	103C8	1560203	g > a	Nonsynonymous	NA	HP1533
Noncoding regions						
24	119A2	472706	c > t		Intergenic	
25	125C7	925414	c > t		Intergenic	

a Position refers to the location of the SNPs in the reference genome of clone H1. HP no. refers to the reference strain H. pylori 26695. ORF, open reading frame; NA, not available.

### Intragenomic rearrangements affecting catalase and lipopolysaccharide (LPS) biosynthesis genes.

**FIG 2:**
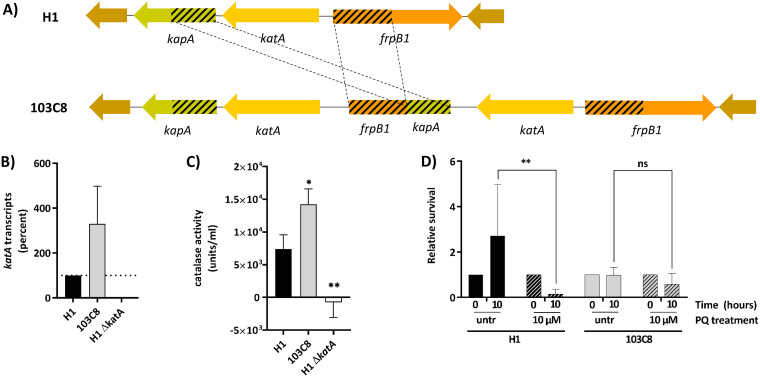
*In vivo* rearrangement in the reisolate 103C8 due to gene fusions and duplication of the *katA* gene. (A) Representation of the genomic context of *katA* in H1 and the duplication of *katA* and a fusion of duplicated fragments of the genes *frpB* and *kapA* in reisolate 103C8. (B) Transcription of the catalase gene in various H. pylori clones was measured by qPCR. *katA* transcript is shown as % relative to the H1 reference, which was set to 100%. The reisolate 103C8 with two *katA* copies showed significantly higher *katA* transcript amounts than H1. A mutant lacking the *katA* gene did not show transcript, as expected. All results were normalized to the respective 16S transcript amounts, also using strain H1 as reference. (C) Catalase activity was measured in bacterial lysates of strain H1, the reisolate 103C8, and H1Δ*katA* (negative control) The reisolate 103C8, with two *katA* copies, displayed higher catalase activity than H1. The calculation of the catalase units/ml is defined as in the Megazyme catalase assay kit instructions. Test, one-way analysis of variance (ANOVA) (*P* < 0.05). (D) Survival experiment using paraquat (PQ) as oxidizing agent on live bacteria. Results are shown as relative survival values, where individual data sets were normalized to their respective bacterial counts obtained at time zero hours, which were set to 1. Strains H1 and 103C8 were treated with 10 μM PQ or left untreated (untr), and the number of colonies was counted 10 h postexposure. Strain H1 was less able to resist the oxidative stress, in contrast to reisolate 103C8, which was more resistant to PQ. Test, two-way ANOVA (*P* < 0.05). For panel B, dotted lines refer to the duplicated and fused regions from the *kapA* and *frpB1* genes. For panels C and D, * = *P* < 0.05; ** = *P* < 0.01; ns, not significant.

The genome of the reisolate 103C8 contained an insertion of 3,433 bp that was initially identified by SMRT sequencing and then confirmed by PCR. The inserted 3-kb fragment contained a second copy of the catalase gene (*katA*, *hp0875*, Rapid Annotation Server [RAST]: fig|210.1715.peg.852) and a fusion of duplicated fragments of the genes *frpB* and *kapA* (Fig. 2A). Since catalase plays an important role in the detoxification of oxygen radicals, we tested whether this duplication had phenotypical effects. Catalase gene transcript was measured by quantitative PCR (qPCR), and it was significantly higher in the reisolate 103C8 than the wild-type strain (Fig. 2B). We measured catalase activity in the respective bacterial lysates, demonstrating that catalase activity in the reisolate 103C8 with two *katA* copies was approximately twice that of the challenge strain (Fig. 2C). We hypothesized that increased catalase activity might help with the detoxification of oxygen radicals and serve to defend against oxidative stress. Thus, the sensitivity to oxidative stress was also comparatively tested for both strains, using paraquat (PQ) as oxidizing agent. The subclone H1 displayed a significant growth delay when exposed to 10 μM PQ, while growth of the 103C8 reisolate was unaffected under the same conditions (Fig. 2D). This result strongly suggests that a second *katA* copy confers a selective advantage to the reisolate 103C8 under conditions of oxidative stress.

Genome analysis of the reisolate 29C8 identified a CNP within a gene encoding an LPS biosynthesis glycosyltransferase (*jhp0562* homolog). This glycosyltransferase was previously shown to be involved in type 1 and type 2 Lewis antigen synthesis (33). Moreover, the presence of this gene was positively associated with the presence of several virulence factors including the *cag*PAI, *vacA*, and several adhesins and has been linked with a higher incidence of peptic ulcers in children (34). The CNP included six synonymous and two nonsynonymous SNPs compared with H1 to H16. Since a recombination with an unrelated strain of H. pylori was quite unlikely in the short-term infection experiment, we performed a genome alignment between the *jhp0562* homolog and its paralogous downstream gene, coding for the *β-*(1,3)-galactosyltransferase [β*-*(1,3) *galT*]. The CNP sequence in the *jhp0562* homolog was identical to the corresponding region in the paralog *galT* (*jhp0563*), suggesting that this CNP was generated by an intragenomic recombination event (Fig. S1A and B). Intragenomic rearrangements between these two genes were in fact described previously (33).

10.1128/mBio.01803-20.1 FIG S1 Intragenomic recombination event between the *jhp0562* homolog and the paralogous gene downstream, *galT*, in the isolate 29C8. (a) Genomic representation of the two genes in the isolate 29C8 and the strain H1. The light blue area represents the CNP occurring in 29C8. (b) The alignment of part of the two genes from H1 and 29C8, where the CNP is located, is shown. The CNP is present in the *jhp0562* homolog gene from 29C8 and in the *jhp0563* homolog gene from H1 and 29C8, suggesting intragenomic recombination. Download FIG S1, PDF file, 0.5 MB. Copyright © 2020 Estibariz et al. 2020 Estibariz et al. This content is distributed under the terms of the Creative Commons Attribution 4.0 International license.

### Diversification of the Opp oligopeptide ABC transporter system.

**FIG 3:**
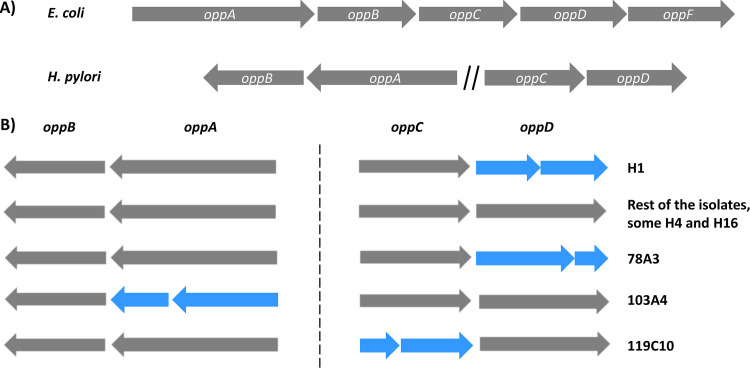
Graphical representation of the H. pylori oligopeptide transport (*opp*) gene cluster and genetic changes observed in BCS 100 and reisolates from infected volunteers. (A) Schematic representation of the *opp* gene clusters in E. coli and H. pylori*. opp* genes form a contiguous gene cluster in E. coli, while the four *opp* genes in H. pylori are located in two different loci. H. pylori does not have a homolog of *oppF*. (B) Graphical representation of the *opp* gene configurations found in strains whose genomes were sequenced in this study. Based on the nucleotide sequences, genes in blue are predicted to be truncated while genes in gray are likely to be active. Note that all reisolates and challenge strain clones H2 to H16 are predicted to express all four *opp* genes.

The oligopeptide transport system (Opp) is typically encoded by five genes in Gram-negative bacteria: the periplasmic oligopeptide-binding protein (*oppA*), two permeases (*oppB* and *oppC*), and two ATP-binding subunits (*oppD* and *oppF*) (35). In H. pylori, the genes belonging to the Opp system are located in two different genomic clusters, *oppA-oppB* and *oppC-oppD*. No homolog for the *oppF* gene has been identified until now (Fig. 3A).

Comparing the *opp* loci between reisolates and challenge strain, we discovered four nonsynonymous SNPs and one SNP introducing a stop codon affecting the genes of the ABC transporter for oligopeptides in four different reisolates. Frameshift mutations leading to a truncation of one of the open reading frames were observed in two more reisolates (Table S4A and Fig. 3B). The gene *oppD* in H1 contains a homopolymeric tract of six adenine residues, leading to a truncated protein. In contrast, the H2 to H16 clones of the challenge strain and all 20 reisolates had seven adenines within the same homopolymeric tract, resulting in a predicted full-length OppD protein. The *oppD* gene of reisolate 78A3 contained a premature stop codon unrelated to the homopolymeric tract. Moreover, we observed several modifications affecting the *opp* genes within the BCS 100 strain, including insertions of 57 bp in the *oppD* gene of H4 and 633 bp within the *oppA* gene of H16, which caused the truncation of the reading frame in H16. Using a combination of primers, we found two differently sized bands when the *oppD* and *oppA* genes were amplified by PCR in clones H4 and H16 (data not shown). We picked 25 colonies from H4 and H16, and using the same combination of primers, we observed that single colonies carried either the insertion or the wild-type allele. Thus, we found that clones H4 and H16 of the BCS 100 challenge strain had developed heterogeneous *oppD* and *oppA* loci due to an unknown mechanism, despite being the result of single colony purification.

In order to evaluate the species-wide variation in genotype of the *opp* gene clusters, we extracted the *opp* gene sequences of 75 representative H. pylori whole-genome sequences from the NCBI database. The four genes were present in all 75 H. pylori strains. Based on the gene sequence, all four *opp* genes were predicted to be functional in 58/75 strains. Putatively inactive *oppA*, *oppC*, and *oppD* genes were observed in 13.33%, 9.33%, and 2.66% of strains, respectively (Table S4B). In contrast, the gene sequence of *oppB* was complete in all the genomes, suggesting an important and conserved role of this gene.

### Selective pressure on the *babA* adhesin gene during early-stage human infection.

Several studies have shown that OMP-encoding genes exhibit sequence changes at higher frequencies than housekeeping genes during the course of H. pylori infection (14, 19, 36). In line with these previous observations, we observed several non-unique CNPs and SNPs affecting OMP-related genes. One of the genes most affected by genomic changes was *babA*, the gene encoding the well-characterized adhesin binding to the Lewis b (Le^b^) histo-blood group antigen (37, 38) (Table S5). Three reisolates carried unique nonsynonymous SNPs or a premature stop codon in *babA*. In addition, a deletion of approximately 1 kb was found in reisolate 29C8 (Fig. S2A). Although BabA expression was confirmed by Western blotting in H1, a Le^b^-binding assay showed that the H1 strain and a representative subset of the reisolates (8A3, 8C10, 12A3, 12C8, 29A2, 29C8, 48A2, and 48C8) were not able to bind Le^b^ compared to the strain J99, which is capable of binding (Fig. S2B).

10.1128/mBio.01803-20.2 FIG S2 Deletion of >1 kb of *babA* in the isolate 29C8 and BabA binding to Le^b^. (a) Genomic context of *babA* in H1 and 29C8 and confirmation of the deletion of approximately 1 kb in *babA* of 29C8 by PCR using specific primers. M, 1-kb marker. The black arrows in the graphic representation of the genes refer to the binding site of the primers. (b) Detection of BabA via WB using whole-cell extracts from H1 and Le^b^ binding of H1, reisolates (8A3, 8C10, 12A3, 12C8, 29A2, 29C8, 48A2, and 48C8), and J99 (used as positive control). The binding was determined by enzyme-linked immunosorbent assay (ELISA). The ratio of binding was calculated as BSA-Le^b^/BSA ratio, where the binding of J99 was set at 100%. Download FIG S2, PDF file, 0.6 MB. Copyright © 2020 Estibariz et al. 2020 Estibariz et al. This content is distributed under the terms of the Creative Commons Attribution 4.0 International license.

### Complete methylome analysis of challenge strain BCS 100 clone H1 and reisolates.

Every H. pylori strain carries a large number of active R-M systems that differ from the R-M set of other strains, leading to strain-specific methylomes (39–41). The plasticity of the methylome during early-phase infection of a new host is still not well understood.

We comparatively analyzed the methylomes of reference challenge clone H1 and all the reisolates using SMRT sequencing technology. In total, 20 different methylated motifs were detected in our set of sequences. Nineteen of these were detected as methylated in H1, and 18 motifs were methylated in H1 and all the reisolates (Table 3 and Table S6). The methylation status of the remaining two motifs (CY^m6^AN_6_TRG and CCA^m6^AK) varied between isolates, suggesting phase variation of the responsible MTase genes. The CYAN_6_TRG motif was methylated in H1 and in 14 of the reisolates. This motif was not detected as methylated in reisolates 12A3, 12C8, 48C8, 81A1, 81C9, and 87C7, the group of isolates that derive from a common ancestor not represented by clones H1 to H16. The motif CCAAK was detected as methylated in only four reisolates (12A3, 12C8, 81A1, and 81C9) and not in H1 or the rest of the reisolates.

**TABLE 3:** Methylated sequence motifs detected by SMRT sequencing in clone H1 of challenge strain BCS 100 and reisolates from human volunteers^*a*^

MTase specificity	Modified base	% motifs detected	No. of motifs in genome	H1#	12A3	12C8	29C8	48C8	78A3	81A1	81C9	87C7
G**A** TC	^m6^A	100	9,890	+	+	+	+	+	+	+	+	+
G**A**NTC	^m6^A	100	5,202	+	+	+	+	+	+	+	+	+
TCNG**A**	^m6^A	100	2,412	+	+	+	+	+	+	+	+	+
GAAG**A**	^m6^A	100	4,446	+	+	+	+	+	+	+	+	+
T**C**TTC	^m4^C	99.98	4,446	+	+	+	+	+	+	+	+	+
TCNNG**A**	^m6^A	100	3,732	+	+	+	+	+	+	+	+	+
C**A** TG	^m6^A	100	14,130	+	+	+	+	+	+	+	+	+
**C**TNAG	^m4^C	100	5,444	+	+	+	+	+	+	+	+	+
GTNNA**C**	^m6^A	100	572	+	+	+	+	+	+	+	+	+
*VCGR****A****G*	^m6^A	100	1,449	+	+	+	+	+	+	+	+	+
*GCRC****A***	^m6^A	100	4,762	+	+	+	+	+	+	+	+	+
TCG**A**	^m6^A	100	530	+	+	+	+	+	+	+	+	+
TGC**A**	^m6^A	99.98	11,346	+	+	+	+	+	+	+	+	+
**C**CGG	^m4^C	99.94	3,358	+	+	+	+	+	+	+	+	+
G**A**GG	^m6^A	99.89	4,494	+	+	+	+	+	+	+	+	+
CY**A**N_6_ TRG	^m6^A	99.87	3,712	+	−	−	+	−	+	−	−	−
**A**TTAAT	^m6^A	99.53	856	+	+	+	+	+	+	+	+	+
GT**A**C	^m6^A	100	198	+	+	+	+	+	+	+	+	+
*GGCA****A***	^m6^A	100	3,364	+	+	+	+	+	+	+	+	+
*CCA****A****K*	^m6^A	99.95	6,357	−	+	+	−	−	−	+	+	−
*CG**C**GCNY	^m4^C			−	+	−	+	−	+	−	−	−
**TGCAGA	^m6^A			−	−	−	+	−	−	−	−	−

a The % of motifs detected and the total number of motifs in the genome are based on the methylome of clone H1. The CCAAK motif quantitation is based on the 12C8 sequence, since the motif was not methylated in clone H1. + means methylation, − means absence of methylation, novel motifs are indicated in italic, and phase-variable MTases are shaded in gray. Modified bases are in bold. Underlined bases refer to the modified base in the complementary strand. *, the GCGC motif that cannot be reliably detected with SMRT sequencing. **, the motif TGCAGA was found only in 29C8 (62.78% of the motifs detected, 309 motifs within the genome), and it might probably be the motif TGCA. #, the following reisolates had methylation patterns identical to strain H1: 8A3, 8C10, 29A2, 48A2, 78C8, 87A3, 103A4, 103C8, 119A2, 119C10, 125A3, and 125C7.

Using the REBASE database (42) and homology-based prediction analysis, we were able to assign 16 motifs to already-known H. pylori MTases (Table S6). While ^m6^A and ^m4^C methylation can be detected by SMRT sequencing, a similarly reliable detection of ^m5^C modifications by SMRT sequencing is not possible (43). The genome of H1 contains genes coding for homologs of three known ^m5^C MTases in H. pylori, and we used restriction analysis using HhaI, HaeIII, and HpyCH4IV restriction enzymes to test the methylation status of predicted ^m5^C motifs. The data confirmed methylation of the G^m5^CGC, GG^m5^CC, and A^m5^CGT motifs in the genome of H1 and likely in the rest of the reisolates since there were no differences in the relevant MTase sequences (Fig. S3). The CCTC and the G^m6^AGG motifs are methylated by two MTases that belong to the same type IIS system, one ^m5^C and one ^m6^A MTase, respectively. The G^m6^AGG motif was detected as methylated in H1 (Fig. S3) and all reisolates, and the fact that both MTase genes of this R-M system are intact (i.e., do not contain premature stop codons) suggests that the motif CCTC is methylated as well.

10.1128/mBio.01803-20.3 FIG S3 Restriction analysis of H1 genome using commercially available restriction endonucleases. Isolated gDNA from the strain H1 was digested (+) with different restriction enzymes. gDNA without enzyme (−) was used as negative control. H1 was resistant to all the tested enzymes as predicted. L, 1-kb ladder. *, BccI enzyme was used as control since H1 is sensitive to cleavage by this enzyme. **, the DpnI enzyme cleaves methylated GATC motifs. ***, the MboI enzyme cuts unmethylated GATC motifs. Download FIG S3, PDF file, 0.5 MB. Copyright © 2020 Estibariz et al. 2020 Estibariz et al. This content is distributed under the terms of the Creative Commons Attribution 4.0 International license.

**FIG 4:**
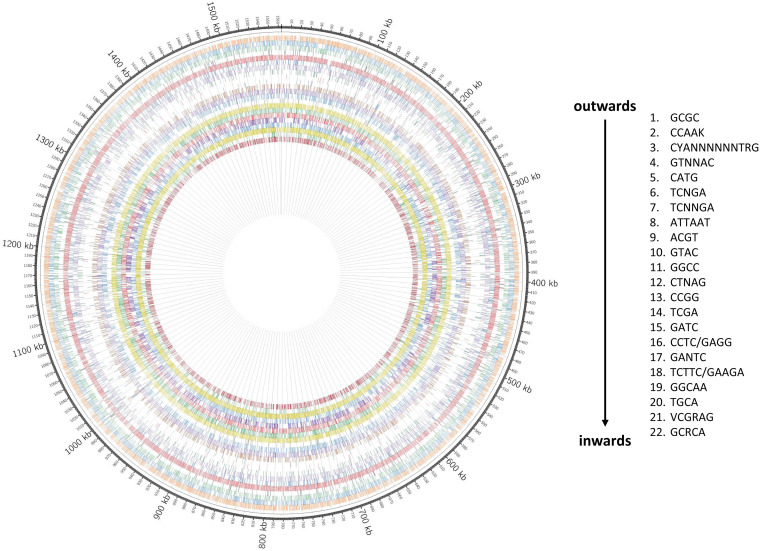
Circos plot displaying the distribution of methylated sequence motifs in H. pylori BCS 100 clone H1. Every circle of colored tracks represents one MTase target motif; the legend at the right side of the figure states the order of motifs represented by the 22 rings, from outer to inner circle. The motif CAAK, which is methylated in some reisolates, is not depicted, because it is not methylated in clone H1.

Accordingly, we identified 24 methylated motifs corresponding to 22 active type II and type III R-M systems (Fig. 4, Table 3, and Table S6). As stated above, the BCS 100 genome contains a predicted type I R-M system that contained multiple sequence polymorphisms, but its target motif and activity status remain unknown. Based on the gene sequences, clones H2 to H16 of the challenge strain are predicted to have the same active MTases as reference clone H1.

Two novel motifs that had not been described before (CCA^m6^AK and GCRC^m6^A) and two motifs that had not yet been assigned to MTase genes but had been detected previously in other H. pylori strains, GGCA^m6^A (44) and VCGR^m6^AG (the motif appears as methylated in the REBASE), were found in this study. Inactivation of selected candidate genes (classified as “genes with unknown function” in our RAST annotation) via insertion of an antibiotic cassette and subsequent SMRT sequencing allowed us to discover three novel MTase genes responsible for the methylation of CCA^m6^AK, GCRC^m6^A, and GGCA^m6^A, respectively. Complete loss of methylation occurred in the mutants with inactivated MTases. None of the other candidate genes tested were responsible for the methylation of VCGR^m6^AG. The MTase responsible for the methylation of this motif thus remains unknown.

The novel MTase (M.HpyH1I) methylating CCA^m6^AK is a phase-variable type III enzyme that contains one homopolymeric tract with 13 guanine residues in frame. The motif was methylated only in the reisolates 12A3, 12C8, 81A1, and 81C9. Sanger sequencing was performed to confirm the number of guanines for all strains. The four reisolates containing methylated CCAAK motifs had an intact open reading frame, while all other reisolates had either eight, nine, or 12 guanine residues, resulting in split open reading frames (Fig. S4A).

10.1128/mBio.01803-20.4 FIG S4 Number of nucleotides within the homopolymeric tracts of the phase-variable R-M systems and graphical representation. (a) R-M system methylating the motif CCAAK. The reisolates with 13 Gs within the homopolymeric tract (highlighted in blue) have an active R-M system. (b) Graphical representation of the R-M system and the S subunit methylating CYANNNNNNTRG. The reisolates with 13 Gs within the homopolymeric tract do not have an active R-M system, and therefore, the CYAN6TRG motif is not methylated in those reisolates (green in the panel). Download FIG S4, PDF file, 0.4 MB. Copyright © 2020 Estibariz et al. 2020 Estibariz et al. This content is distributed under the terms of the Creative Commons Attribution 4.0 International license.

A second phase-variable R-M system (HpyH1III) methylating the motif CY^m6^AN_6_TRG was found. The MTase in charge was identified by homology. This motif was methylated in 14 reisolates and in reference clone H1 but not in the reisolates 12A3, 12C8, 48C8, 81A1, 81C9, and 87C7. The status of the homopolymeric tract was checked by Sanger sequencing and was in complete agreement with the methylation patterns in all strains (Fig. S4B). One homologous enzyme has been described in H. pylori UM032 (44). Lee and colleagues discovered that a 12-guanine homopolymeric tract split the S subunit in two (S1 and S2), and the expression of S1 led to methylation of the motif.

## DISCUSSION

Due to its high mutation and recombination rates and the resulting very high level of within-species genetic diversity, H. pylori has become a paradigm for the within-host evolution of bacterial pathogens. Most studies of the *in vivo* evolution of the H. pylori genome were performed with sequential isolates from chronically infected adult patients. In contrast, little is yet known about the genome evolution of H. pylori during the initial phase of the infection, when selection is likely to be strongest due to the need for the bacteria to establish infection in a new host after a massive bottleneck caused by the transmission (13, 45, 46). Furthermore, the methylomes of H. pylori are highly complex, and so far, the role of epigenetic modifications in the adaptation of this gastric pathogen to a new stomach niche or new hosts is not known. To our knowledge, only one previous study from our group has described the *in vivo* methylome adaptation during acute infection, where methylome variation during infection was observed as a result of phase variability in MTase genes (20). In the present study, we report the genome and methylome evolution of H. pylori after a short-term infection of human volunteers with a *cag*PAI-negative strain, BCS 100. In contrast to the previous study, reisolates from two different stomach locations from each volunteer were available for analysis.

All H. pylori strains cultured from unrelated individuals display specific genotypic and phenotypic characteristics. There is also extensive intrastrain heterogeneity, such that H. pylori has been termed a “quasispecies” (47). Human volunteer infection studies are particularly informative about genome-based adaptation to a new host, since the duration of infection is precisely known, and both ancestral and evolved strains can be characterized in depth. In the present work, the analysis of 16 purified single colonies from the challenge strain BCS 100 showed that it is not genetically homogeneous. In addition to differences among the 16 clones purified from the challenge strain population, intraclonal variability within some of the single colony-purified clones of the BCS 100 strain was detected, including insertions and deletions within *opp* genes. Our observation of genetic heterogeneity within BCS 100 is in agreement with a previous study (19), where diversity at six loci selected from a draft genome comparison between clone H1 and a single reisolate (8A3) corresponded to nucleotide polymorphisms now found to be present in clones H1 to H16. In addition, the observation of several non-unique SNPs and CNPs that were detected in multiple reisolates but in none of the clones H1 to H16 provides strong evidence that the challenge strain contained further subpopulations not sampled in clones H1 to H16. Despite the diversity observed in the inoculum, phylogenetic analysis of the challenge strain and the reisolates (Fig. 1B) revealed that eight out of 10 volunteers were likely colonized by the same subpopulation from the inoculum. In two volunteers, paired isolates from the antrum and corpus belonged to distinct subclusters. Both these observations imply that H. pylori populations experience a strong bottleneck after person-to-person transmission. Previous experimental infection studies in humans (29) and in mice (48) suggested that only a small number of bacterial cells are establishing the infection early on. Our data indicate that these initial colonizing clones are also likely to be genetically homogenous, with few exceptions. Consequently, the distinct selective environmental pressures of a new gastric niche might result in a strong reduction of the inoculum diversity during the early stages of infection.

Whole-genome comparisons of the reisolates with the challenge strain allowed us to calculate *in vivo* mutation rates. The calculated mutation rates from our data were in agreement with estimates obtained in previous studies that were performed with sequential isolates from chronically infected individuals, and with one previous study performed on isolates from challenged human volunteers (19, 20, 49). Notably, this is the second independent study performed on reisolates from challenged human volunteers that did not show evidence of a “mutational burst” during early-stage human infection, as described in one study by Linz et al., where two patients were first treated with antibiotics and subsequently reinfected with their own H. pylori strain (50). We noted a very high proportion of nonsynonymous mutations among the unique polymorphisms in the reisolates, which are likely to have evolved *de novo* since first colonization of a new host. Seventy-six percent of the mutations were nonsynonymous (nonsynonymous/synonymous ratio 3.76). This ratio is similar to the ratio of 2.8 observed in the previous study with challenge strain BCM-300 but substantially higher than the ratio of 0.95 observed for four pairs of genomes from sequential H. pylori isolates cultured from chronically infected individuals with a time interval of 3 years (19). It has been well documented that nonsynonymous mutations are more common in populations that have undergone a recent strong expansion (as would have happened after experimental transmission). Many slightly deleterious nonsynonymous mutations are lost from the population with time, as shown for H. pylori by the comparisons between short-term (years [19]) and long-term (millennia) mutation rates (13). However, the fact that in the reisolates of the present study, nonsynonymous mutations were strongly enriched in genes coding for specific functional classes, such as bacterial envelope traits and host interaction molecules, strongly suggests that the observed patterns of mutations reflect early adaptation to the new host rather than a random distribution of near-neutral mutations.

We recently analyzed patterns of genetic diversity in genomes from H. pylori isolates from different regions of the stomach, using 10 isolates per location. This analysis provided evidence that genes involved in chemotaxis, motility, transport, and cellular processes, as well as OMP-related genes, were particularly prone to diversify *in vivo* (“high-frequency host variable genes”) (32). Despite the short time of infection in our present study, several genes that contained unique polymorphisms (*oppB*, *arcS*, *hcpD*, *hp0953*, and *hp0130*) overlap the list of high-frequency host variable genes from the analysis by Ailloud et al. (32), again suggesting that the patterns of diversity observed reflect *in vivo* selection rather than random mutational patterns.

Genes with an outer membrane-related role have been shown to vary at higher frequencies than other genes in H. pylori (14, 19, 51). In agreement with previous findings, our data emphasize that most of the SNPs identified were again in genes encoding outer membrane proteins. Unique SNPs within *babA*, the gene encoding the best-characterized outer membrane protein of H. pylori, the Le^b^ binding adhesin, were detected in several reisolates of this study. One reisolate (29C8) harbored a deletion of approximately 1 kb affecting *babA*. In agreement with this, BabA expression was not detected in this strain. Both frequent mutations and complete loss of BabA expression were reported during infection in primates and humans (37, 52–54). Frequent mutations in *babA* and signs of positive (diversifying) selection have been interpreted as signs of adaptation to individual hosts or different niches within one host (e.g., antrum versus corpus). In the case of strain BCS 100, the frequent changes in *babA* are less easily explained by selection, because clone H1 (or the reisolates) did not exhibit binding to Le^b^ antigen, despite its expression of the adhesin protein BabA. Previously, it was shown that eight amino acids are responsible for the binding to Le^b^ (55). A possible reason for the absence of binding by clone H1 to Le^b^ might be a single amino acid substitution in the amino acid sequence of BabA affecting the Fuc4 binding site. Specifically, Asn206, shown to be involved in Le^b^ binding in strain J99, is substituted by a positively charged Glu in clone H1 and in all derivatives of challenge strain BCS 100. This might explain the general lack of Le^b^ binding of all tested clones of the input and output from this experimental infection. While adhesion is considered a pivotal part of H. pylori pathobiology, and BabA is the by-far best-characterized adhesin (56), it has also long been known that not all strains bind to Le^b^ (57), and the functional role of the variant version of BabA expressed by H1 is unknown. It has been proposed that BabA may serve a still-uncharacterized role explaining the strong selective pressure against expression of BabA in some model systems (54). Our observation that multiple reisolates differed in BabA expression would also be consistent with the hypothesis that BabA might have additional ligands in the absence of Le^b^ binding.

In the same reisolate displaying the partial deletion of *babA* (29C8), we also observed an intragenomic recombination event between two genes described as essential for the synthesis of type 1 and type 2 Lewis antigens in the bacteria. It was previously suggested that recombination between *jhp0562* and the *β-*(1,3) *galT* gene generates Lewis antigen diversity (33). This mechanism has been suggested to be valuable for H. pylori to mimic the human Lewis antigens on its surface in order to evade the host immune system (58, 59). Possibly, strains mimicking the human Le^b^ antigen on their own surface might be prone not to express Le^b^-binding BabA. This hypothesis needs to be further explored.

One of the striking observations in this study was the unexpected frequency of mutations in genes of the oligopeptide ABC transporter system, suggesting selective evolution during the acute infection of human volunteers. Although the genes were originally annotated as part of the oligopeptide uptake system, an additional role in short peptide uptake has been suggested (60). Both oligopeptide (Opp) and dipeptide (Dpp) transport systems may contribute to the import of carbon and nitrogen sources from the human stomach into the bacterium (60, 61). Several other roles, ranging from the recycling of cell wall peptides to host adhesion, have been attributed to the Opp systems in other microorganisms (62). Whether the Opp system in H. pylori similarly has additional functions will have to be addressed in the future, but it seems that the Opp system plays an important role in H. pylori adaptation to a new host, at least in the context of the strain BCS 100. Interestingly, the permease gene *oppB* was the only gene of the *opp* operon predicted to be functional in all H. pylori genomes analyzed here, suggesting an important function of this gene either within the Opp system or in a different process. One plausible explanation for the selection of nonfunctional Opp system components in some reisolates could be the absence of specific nutrients from a specific stomach niche. Inactivation of individual Opp system components might help to alleviate metabolic costs of the expression of Opp transporters. Modifications were not found within the *opp* genes in the reisolates of the infected volunteers challenged with the BCM-300 strain. It was suggested that CagA and VacA might promote colonization in iron-limited environments via facilitating iron acquisition from the host epithelium (63). The authors also proposed that H. pylori must acquire other micronutrients from the host. Thus, it is attractive to speculate that in the absence of bacterial factors like the type 4 secretion system (T4SS) potentially facilitating nutrient acquisition, the bacteria might modulate the uptake of nutrients by altering the activity of permease genes such as the Opp system. However, only 12 reisolates were available in BCM-300, and future studies will be required to test the hypothesis that inflammation status and mutations in *opp* genes might be linked.

The experimental vaccine used in this trial was a *Salmonella* strain expressing H. pylori urease. None of the reisolates showed mutations in genes of the urease operon, so there was no evidence for vaccine-induced immune evasion. This differs from our previous study with challenge strain BCM-300 (20), where multiple reisolates lost the expression of antigens contained in the experimental vaccine (VacA cytotoxin and CagA), possibly at least in part as a result of vaccine-induced immune selection. In contrast with CagA and VacA, urease expression is essential for H. pylori infection, which is one of the reasons why many vaccine studies have used urease as antigen. The BCS 100 strain is *cag*PAI^−^ while the challenge strain BCM-300 used in the other study is *cag*PAI^+^. This difference leads very likely to differences in inflammation in the stomach during short-term infection, which might, in the BCS 100 study, reduce the selective pressure by oxidative stress and the immune response.

Another interesting finding was the duplication of the *katA* gene in the reisolate 103C8. As a result of the duplication of *katA* that had occurred during infection, this reisolate displayed higher catalase activity and was less sensitive to oxidative stress induced by paraquat. Hence, higher catalase production and activity might be beneficial for H. pylori infecting a host with an increased inflammatory response, since such processes release more reactive oxygen species (ROS). Based on the histology data (28), the volunteer 103 from whom reisolate 103C8 was cultured had one of the highest scores of inflammation (gastroscopy, 6 weeks postinfection [wpi]). In addition, H. pylori was suggested to use its catalase activity to survive ROS caused by phagocytosis (64). Since the corresponding antrum reisolate 103A4 did not have a second catalase gene, it seems reasonable to consider that within the H. pylori population infecting this volunteer there was a subpopulation of reisolates that were selected, possibly in the corpus of the volunteer, able to resist higher levels of oxidative stress.

Methylation regulates the expression of several genes allowing the microorganisms to respond rapidly to external signals due to changes in DNA methylation patterns. In the present study, using a combination of SMRT sequencing technology and restriction of genomic DNA (gDNA) with commercially available restriction enzymes, we confirmed 24 methylated motifs in the challenge strain and reisolates. Most of the motifs were assigned to already known MTase specificities, but we found methylated sites not described before (CCA^m6^AK and GCRC^m6^A) or found in other H. pylori strains but not assigned to any MTase (GGCA^m6^A and VCGR^m6^AG). The inactivation of candidate MTase genes and subsequent SMRT sequencing of the mutants allowed us to discover three novel R-M systems. Differences in the methylomes between challenge strain and reisolates were due to phase variation of two R-M systems methylating CCA^m6^AK and CY^m6^AN_6_TRG. To our knowledge, this is the second study showing an *in vivo* switch in the activity of MTase genes in H. pylori during acute or early-stage infection (20). This strongly suggests that the reversible ON/OFF switch of these enzymes confers an adaptive advantage of H. pylori in new niches, possibly via the transcriptional regulation of multiple genes. Phase-variable MTases can contribute to bacterial pathogenesis via modifying the expression of multiple genes that together form a “phasevarion” (phase-variable regulon of genes) (65). Phasevarions were identified in many bacterial pathogens including H. pylori (24–26). The *flaA* gene and the outer membrane protein gene *hopG* were differentially regulated when one yype III MTase, *modH5*, was not active in H. pylori strain P12 (26). The novel phase-variable MTase M.HpyH1I that methylates CCAAK is a homolog of ModH5 in the P12 strain (79.37% nucleotide sequence identity), both located downstream of the ATP-dependent DNA helicase RecG. There are multiple CCAAK motifs in *recG* (both within the coding sequence and immediately upstream of the start codon). Hence, a potential role of CCAAK methylation in the transcriptional control of *recG* and other genes is possible and will be the subject of future studies. In addition, a role for phasevarions in otitis media diseases has been proposed based on the distribution of phase-variable MTases in Moraxella catarrhalis (66). In pathogenic *Neisseria*, phasevarions produce different bacterial populations with distinct abilities in colonization (25). Thus, it is conceivable that the modulation of the expression of several genes by the activity of phase-variable MTase genes can contribute to the adaptation of H. pylori to new niches, and this will be explored in future studies.

In conclusion, human volunteer challenge studies performed in the context of vaccine trials are a powerful approach to study short-term adaptation of pathogens to a new host. One unavoidable limitation of this approach is due to the fact that volunteer challenge studies can be performed only in adults while most natural H. pylori infections occur in children. Using one of two such studies available for H. pylori experimental vaccination, we found that the gastric pathogen H. pylori copes with the adaptation requirements during early infection by altering several genes and their functions. This concerns mostly genes and proteins with a role in surface modulation. However, many reisolates after short-term colonization seem to develop quite different and diverse strategies, via modifying diverse genes and parts of their genomes. This diversity implies that the genetic and phenotypic malleability and ways to adapt in H. pylori are manifold and that various adaptation paths in the face of environmental pressures are possible. Among other mechanisms, global coordinated gene regulation via phasevarions might contribute substantially to the early-stage adaptation of H. pylori to new niches.

## MATERIALS AND METHODS

### Challenge strain and reisolates.

H. pylori strain BCS 100 (ATCC BAA-945) was isolated from an infected individual with mild superficial gastritis and described elsewhere (29). H. pylori BCS 100 was used to challenge human volunteers as a part of a vaccination study, administering an inoculum dose of 2 × 10^5^ bacteria (CFU) (28). Pairs of reisolates from the antrum and corpus location in the stomach of each infected individual were obtained 10 weeks after the infection. The bacterial samples were isolated from volunteers belonging to control or vaccination groups (see Table S1 in the supplemental material). In contrast to classical microbiology technique, input strain BCS 100 was maintained without single-colony purification, conserving within-strain variation. All genome sequences analyzed in this study were obtained from single-colony purified clones. Clones H1 through H16 were single-colony purified from strain BCS 100 at the Max Planck Institute for Infection Biology at the time when the clinical trial was prepared. Cultures used for this study were inoculated using stock cultures made at this time, so that the number of passages since the isolation of the individual clones was kept low, below five. All genome sequences from reisolates were also obtained from single-colony purified clones. For each of the 10 volunteers studied (five volunteers randomly selected from the vaccine and control groups, respectively), one reisolate from the antrum and one from the corpus were analyzed.

10.1128/mBio.01803-20.5 TABLE S1 List of H. pylori reisolates used in this study and histology scores at the time of H. pylori culture from challenged human volunteers (table modified from the work of Aebischer et al. [T. Aebischer, D. Bumann, H. J. Epple, W. Metzger, et al., Gut 57:1065–1072, 2008, https://doi.org/10.1136/gut.2007.145839]). Download Table S1, PDF file, 0.5 MB. Copyright © 2020 Estibariz et al. 2020 Estibariz et al. This content is distributed under the terms of the Creative Commons Attribution 4.0 International license.

### Bacterial strains and culture conditions.

H. pylori strains were cultured on blood agar plates (blood agar base II; Oxoid, Wesel, Germany) containing 10% horse blood and supplemented with antibiotics (vancomycin [10 mg/liter], polymyxin B [3.2 mg/liter], amphotericin B [4 mg/liter], and trimethoprim [5 mg/liter]) as previously described (67). H. pylori liquid cultures were performed in brain heart infusion broth (BHI; BD Difco, Heidelberg, Germany) with yeast extract (2.5 g/liter), 10% heat-inactivated horse serum, and the same combination of antibiotics as on the blood agar plates (23). Escherichia coli strains were grown in LB medium (Lennox L broth; Invitrogen Life Technologies, Darmstadt, Germany) supplemented with ampicillin (200 μg/ml) and/or kanamycin (20 μg/ml) as described previously (41).

### DNA techniques and next-generation sequencing.

All procedures were performed following the manufacturer’s protocols. Genomic DNA (gDNA) was purified using Genomic-tip 100/G columns (Qiagen, Hilden, Germany). The QIAprep Spin Miniprep kit (Qiagen, Hilden, Germany) was utilized for E. coli plasmid isolation.

Complete genome sequencing of clone H1 from the challenge strain BCS 100 and the reisolates was performed using SMRT sequencing technology on a Pacific Biosciences RSII instrument. Preparation of SMRTbell template libraries was carried out as described previously (20). One SMRT cell was sequenced per strain using P6/C4 chemistry. *De novo* genome assemblies were carried out using Hierarchical Genome Assembly Process 3 (“RS_HGAP_Assembly.3” protocol) within SMRT Portal 2.3.0. Complete genomes have been circularized and rotated to the *dnaA* gene as starting position. The standardized “RS_Modification_and_Motif_Analysis.1” protocol was used applying default parameters to detect methylated bases and to identify the motifs as performed in references 20 and 41.

Genome sequences of the strains H2 to H16 were obtained using an Illumina MiSeq system as described before (23). Libraries were prepared using the Nextera DNA sample preparation kit (Illumina, San Diego, CA, USA). The length of the fragments was calculated applying the high-sensitivity DNA analysis kits (Agilent Technologies, Palo Alto, CA, USA) in an Agilent 4200 TapeStation device. Illumina MiSeq 2 × 300 cycle reagent kit v3 was employed to sequence the libraries in a MiSeq sequencer. *De novo* assembly of the paired-end reads was performed using SPAdes 3.9.0 with default parameters (68).

### Genome comparison.

The genome of clone H1 was annotated using the Rapid Annotation Server (RAST). KODON (Applied Maths, Sint-Martens-Latem, Belgium) and Geneious 11.0.2 (69) were used for whole-genome comparison of H1 (used as reference) with the reisolates and clones H2 to H16. As described previously, differences were classified as single nucleotide polymorphisms (SNPs) or as clusters of polymorphisms (CNPs). CNPs consist of a group of polymorphisms within less than 200 contiguous bp that are flanked by at least 200 bp of identical sequence on both sides. CNPs are, from previous evidence, most likely the result of allelic replacement events after homoeologous recombination (12, 19). Phylogenetic inference was performed with *M*_r_ Bayes 3.2 (70), using 1,000,000 generations and 10% burn-in.

### Selection and inactivation of candidate MTase genes and graphical representation.

Identification of R-M genes within the genome of reference clone H1 was performed as described in reference 71. Using the REBASE database (42), we predicted the specificities of previously described MTase genes and selected candidate genes encoding putative MTases. The candidate genes were inactivated via insertion of an *aphA3* cassette conferring resistance to kanamycin as described previously (41). Plasmids containing the interrupted gene were used for natural transformations using H. pylori clone H1 or the reisolate 12C8 as recipient. Successful allelic exchange between the plasmid and the chromosomal target gene was confirmed by PCR. All primers and strains used are listed in Table S2A and B, respectively.

10.1128/mBio.01803-20.6 TABLE S2 Oligonucleotides (A) and recombinant bacterial strains (B) used in this study. Download Table S2, PDF file, 0.1 MB. Copyright © 2020 Estibariz et al. 2020 Estibariz et al. This content is distributed under the terms of the Creative Commons Attribution 4.0 International license.

10.1128/mBio.01803-20.7 TABLE S3 SNPs (A), and CNPs (B) within the BCS 100 strain (clones H1 to H16), and CNPs within the reisolates (C). The SMRT-sequenced genome of clone H1 was used as a reference for the genome alignments. Download Table S3, PDF file, 0.4 MB. Copyright © 2020 Estibariz et al. 2020 Estibariz et al. This content is distributed under the terms of the Creative Commons Attribution 4.0 International license.

10.1128/mBio.01803-20.8 TABLE S4 Modifications affecting Opp system components within BCS 100 (clones H1 to H16) and reisolates (A) and *opp* gene status in 75 diverse H. pylori genomes (B). Download Table S4, PDF file, 0.6 MB. Copyright © 2020 Estibariz et al. 2020 Estibariz et al. This content is distributed under the terms of the Creative Commons Attribution 4.0 International license.

10.1128/mBio.01803-20.9 TABLE S5 Genetic changes in adhesin gene *babA* observed in the reisolates from the different volunteers compared to the H1 to H16 clones. Download Table S5, PDF file, 0.5 MB. Copyright © 2020 Estibariz et al. 2020 Estibariz et al. This content is distributed under the terms of the Creative Commons Attribution 4.0 International license.

10.1128/mBio.01803-20.10 TABLE S6 MTase genes in strain BCS 100 and reisolates, and their cognate REase and S subunit genes. Download Table S6, PDF file, 0.5 MB. Copyright © 2020 Estibariz et al. 2020 Estibariz et al. This content is distributed under the terms of the Creative Commons Attribution 4.0 International license.

Graphical representation of motifs within the H1 genome was generated using Circos (72) (Fig. 4).

### Restriction assays.

Isolated H. pylori gDNA (250 to 300 ng) was used in a 20-μl reaction mixture containing the corresponding amount of restriction enzyme buffer (NEB, Frankfurt am Main, Germany), 1 μl of the suitable restriction enzyme (NEB, Frankfurt am Main, Germany), and high-pressure liquid chromatography (HPLC) water. A 20-μl sample with the same amount of gDNA, restriction enzyme buffer, and HPLC water without enzyme was used as negative control. Both tubes were incubated at 37°C for 1 h. Then, 10 μl of the reactions was loaded onto a 1% agarose gel to detect the digested/undigested gDNA patterns.

### Isolation and detection of proteins.

H. pylori from 22- to 24-h blood agar plate cultures was harvested in 1 ml cold phosphate-buffered saline (PBS) (600 × *g*, 4°C, 5 min). Cell pellets were suspended in 200 μl of homogenization buffer (Tris-HCl, 100 mM, pH 7.4). Samples were sonicated for 1 min at continuous pulse. Total protein concentration was calculated using the Pierce bicinchoninic acid (BCA) protein assay kit (Thermo Fisher Scientific, Darmstadt, Germany). The same amounts of protein samples were separated on SDS-PAGE gels (14%). Subsequently, Western blotting (WB) was performed for the detection of target proteins. Detection of BabA was achieved with a rabbit antiserum kindly provided by Thomas Borén (Umeå University, Sweden) and a secondary peroxidase-labeled Affini-pure goat anti-rabbit IgG (H+L) antibody (Jackson Immuno Research Laboratories, USA). Catalase detection was achieved using an antibody from the Ridascreen FemtoLab stool antigen test (R-Biopharm AG, Darmstadt, Germany) as described previously (8).

SuperSignal West Pico chemiluminescent substrate (Thermo Fisher Scientific, Darmstadt, Germany) was used for WB developing. The Las-3000 imaging system (Fujifilm Life Science, Düsseldorf, Germany) and chemiluminescence were used to reveal the WB.

### qPCR.

Quantitative PCR (qPCR) was performed as described before (73). Synthesis of cDNA was performed with SuperScript III reverse transcriptase (Thermo Fisher Scientific, Darmstadt, Germany) using 1 μg of RNA. A Bio-Rad CFX96 system was used to perform the qPCR with specific primers (Table S2A) and SYBR green Master Mix (Qiagen, Hilden, Germany). Samples were normalized to an internal 16S rRNA control. Reaction conditions in agreement with the MIQE (minimum information for publication of quantitative real-time PCR experiments) guidelines are available in the supplemental material. GraphPad Prism 7 (GraphPad Software, La Jolla, CA, USA) was used to compile all graphs.

### Oxidative stress assay.

The oxidative stress assay was carried out as described previously (74). Liquid cultures were inoculated with challenge strain clone H1 or with the reisolate 103C8 and grown overnight (37°C, 175 rpm, microaerobic conditions, initial optical density at 600 nm [OD_600_] = 0.06). Afterward, 10 μM paraquat (PQ) (paraquat dichloride hydrate [Pestanal], number 36541; Sigma-Aldrich, Germany) was added to the cultures; controls were left untreated. The time point of the inoculation was counted as time zero. Serial dilutions were plated onto blood agar plates at time zero and after 10 h. The plates were incubated at 37°C and under microaerobic conditions. Finally, after 4 to 5 days of incubation, the number of colonies was counted. Data were normalized to time point 0.

### Catalase activity.

Catalase activity was determined using the Megazyme catalase assay kit (Megazyme, Butzbach, Germany) following the manufacturer’s instructions. Bacteria from 20 to 22-h blood agar plate cultures were harvested and suspended in ice-cold 1× PBS to an OD_600_ of 1. Lysates were obtained as mentioned above, and 1:1,000 dilutions of protein lysates were used to perform the assays. Three independent biological replicates were carried out for each sample.

### Le^b^ binding assay.

The Le^b^ binding assay was carried out as described previously (20), with minor modifications. Bacteria from blood agar plates (incubated for 22 to 24 h) were suspended in 1 ml PBS and centrifuged (2,800 × *g*, 5 min, 4°C). The OD_600_ was measured and adjusted to 2 × 10^8^ cells in 450 μl of PBS for subsequent biotinylation during 1 h with N-hydroxysuccinimide-long chain (NHS-LC) biotin (succinimidyl-6-(biotinamido)hexanoate, Thermo Fisher Scientific, Darmstadt, Germany) (125 μg/25 μl). Then, 250 ng of bovine serum albumin (BSA) or 250 ng of Le^b^-BSA was used to coat a 96-well covalent microtiter plate (Corning Costar, USA). Immobilization of the glycoproteins was performed under UV light for 30 s, and afterward, the plate was blocked with PBS containing 5% BSA. Subsequently, 50 μl per well of biotinylated bacteria was coincubated in the dark for 1 h and washed 3 times with PBS. Sample fixation was achieved with 100 μl of 2% paraformaldehyde (100 mM potassium phosphate buffer, neutral pH). The plate was washed 3 times with wash buffer (0.05% Tween 20 in PBS), blocked for 1 h with assay diluent (PBS containing 10% fetal calf serum), and again washed 5 times with wash buffer. After an incubation step of 90 min at room temperature with neutravidin-horseradish peroxidase (HRP) conjugate in assay diluent, the plate was rinsed again and incubated with tetramethylbenzidine (TMB) substrate (BD Biosciences). Using 50 μl per well of 1 M H_3_PO_4_, the reaction was stopped. The signal was detected in a microplate reader at 450 nm. Only one replicate per sample (in technical triplicates) was performed since no binding was detected for the strain H1 while a clear binding was determined for the positive control, strain J99.

### Data availability.

Sequence data have been deposited in the NCBI with link to BioProject accession PRJNA522954 (https://www.ncbi.nlm.nih.gov/bioproject/PRJNA522954).
