# Supplementary material for: In Vivo Genome and Methylome Adaptation of cag-Negative Helicobacter pylori during Experimental Human Infection
Source: mBio. 2020 Aug 25;11(4):e01803-20. doi: 10.1128/mBio.01803-20 (PMC7448279; doi:10.1128/mBio.01803-20)
Supplement: FIG S1 [file mBio.01803-20-sf001.pdf]

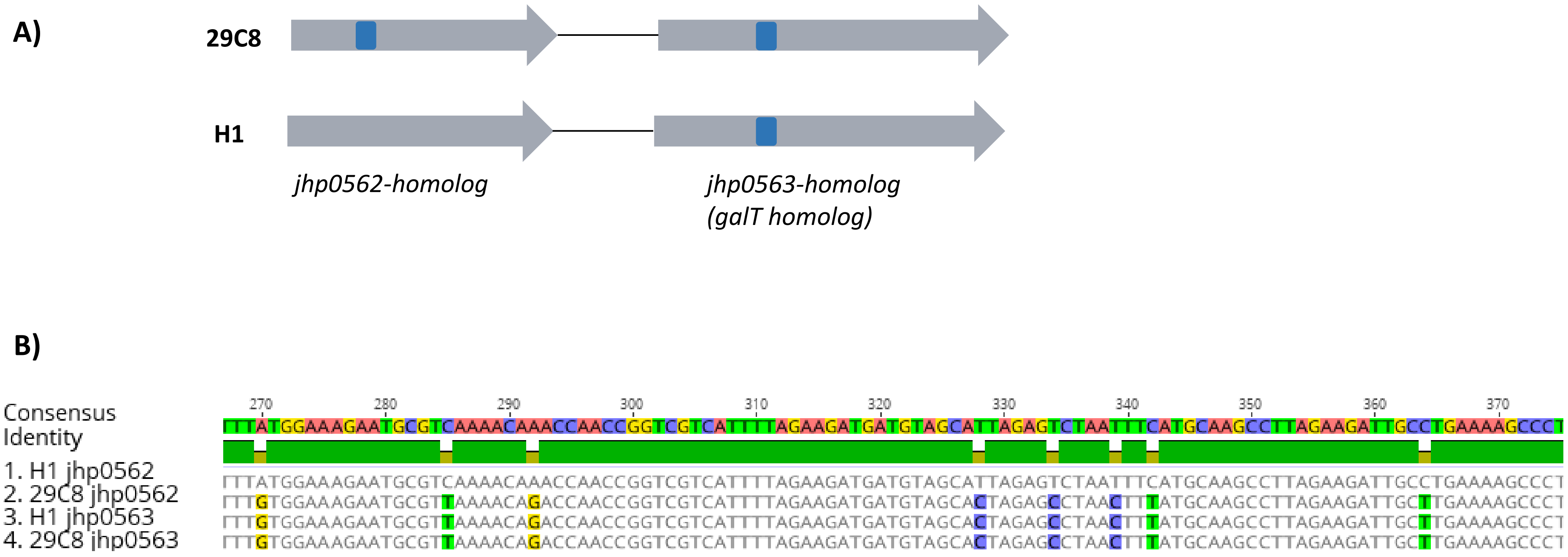

Figure S1. Intragenomic recombination event between the *jhp0562* homolog and the paralogous gene downstream, *galT*, in the isolate 29C8**.** (a) Genomic representation of the two genes in the isolate 29C8 and the strain H1. The light blue area represents the CNP occurring in 29C8. (b) The alignment of part of the two genes from H1 and 29C8, were the CNP is located, is shown. The CNP is present in the *jhp0562*-homolog gene from 29C8 and in the *jhp0563*-homolog gene from H1 and 29C8, suggesting intragenomic recombination.
