# Supplementary material for: In Vivo Genome and Methylome Adaptation of cag-Negative Helicobacter pylori during Experimental Human Infection
Source: mBio. 2020 Aug 25;11(4):e01803-20. doi: 10.1128/mBio.01803-20 (PMC7448279; doi:10.1128/mBio.01803-20)
Supplement: FIG S2 [file mBio.01803-20-sf002.pdf]

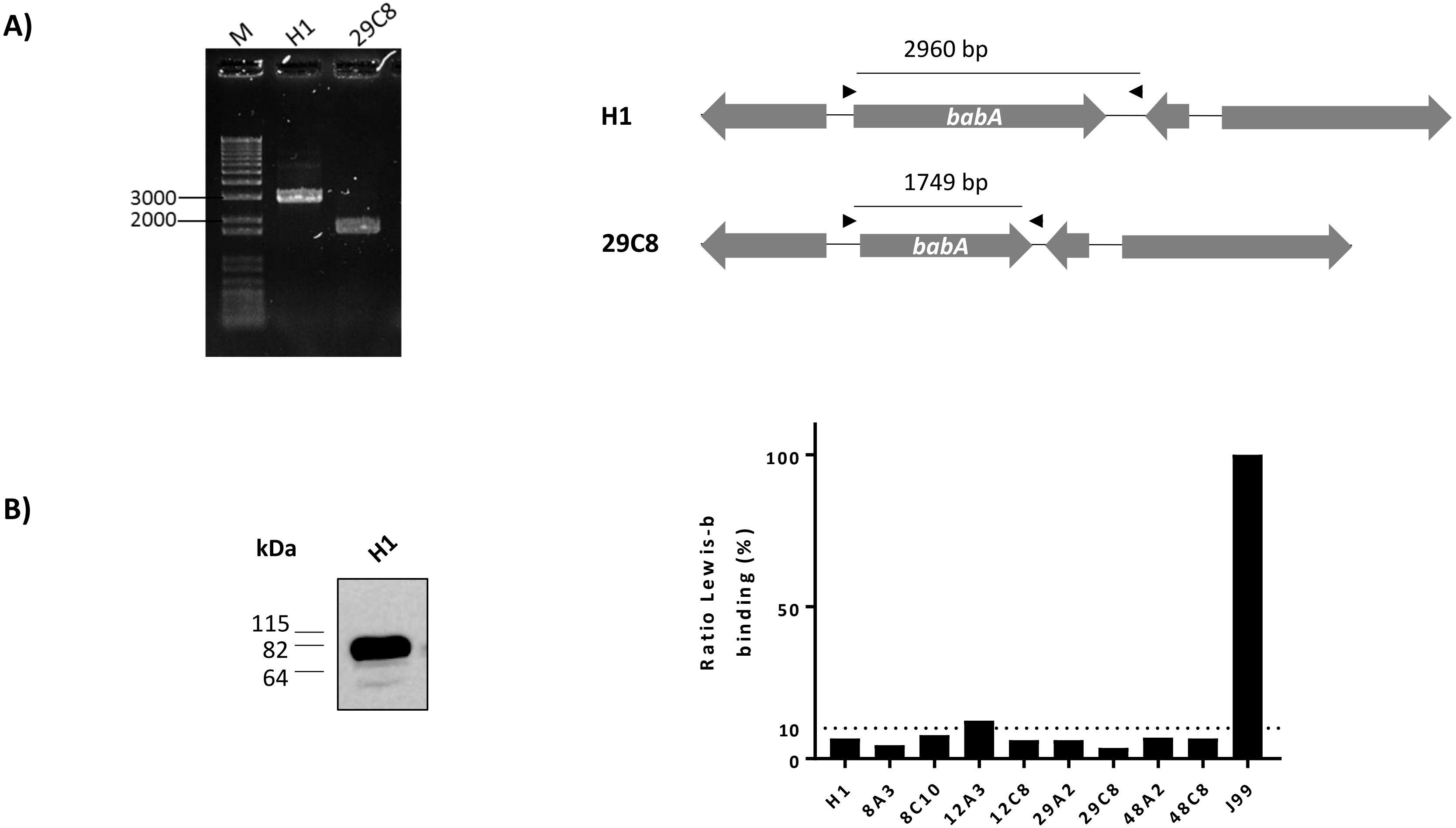

Figure S2. Deletion of >1 kb of *babA* in the isolate 29C8 and BabA binding to Le(b). (a) Genomic context of *babA* in H1 and 29C8 and confirmation of the deletion of approximately 1 kb in *babA* of 29C8 by PCR using specific primers, M: 1 kb marker. The black arrows in the graphic representation of the genes refer to the binding site of the primers. (b) Detection of BabA via WB using whole cell extracts from H1, and Le (b) binding of the H1 and reisolates (8A3, 8C10, 12A3, 12C8, 29A2, 29C8, 48A2 and 48C8) and J99 (used as positive control). The binding was determined by ELISA. The ratio of binding was calculated as BSA-Le (b)/BSA ratio, where the binding of J99 was set at 100%.
