# Supplementary material for: In Vivo Genome and Methylome Adaptation of cag-Negative Helicobacter pylori during Experimental Human Infection
Source: mBio. 2020 Aug 25;11(4):e01803-20. doi: 10.1128/mBio.01803-20 (PMC7448279; doi:10.1128/mBio.01803-20)
Supplement: FIG S3 [file mBio.01803-20-sf003.pdf]

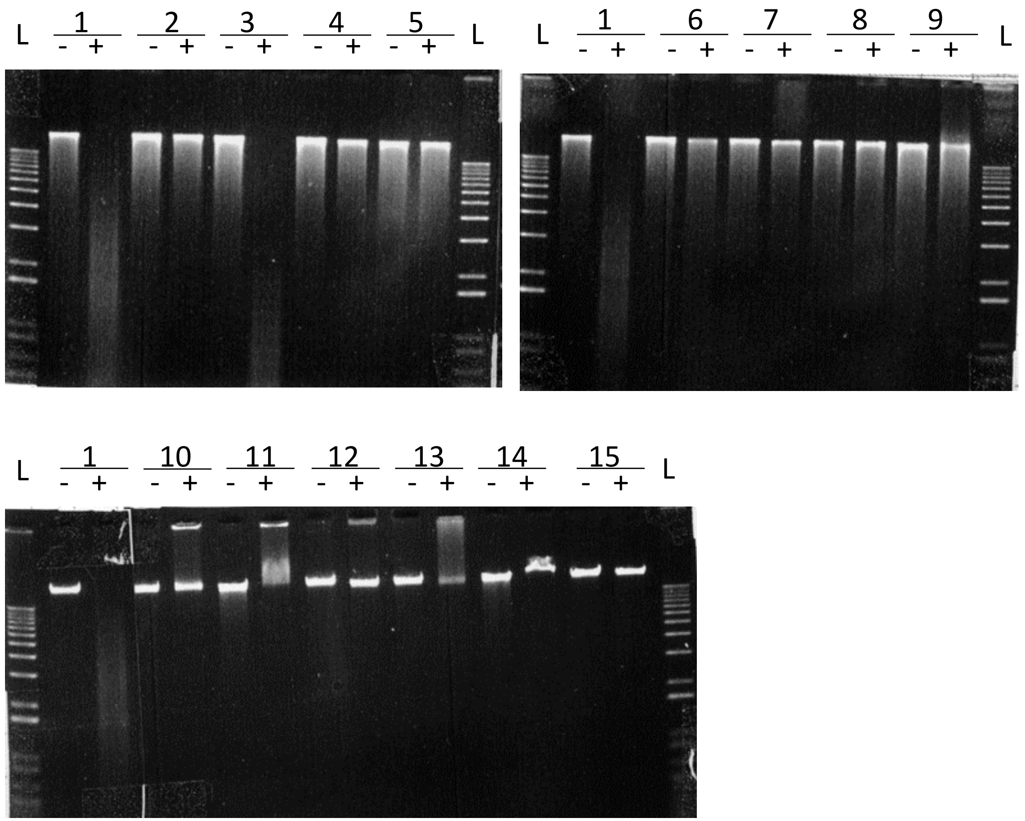

N°	Enzyme	Motif
1*	BccI	CCATC
2	HinfA	GANTC
3**	DpnI	GATC
4***	MboI	GATC
5	Hpy188III	TCNNGA
6	HhaI	GCGC
7	Hpy166II	GTNNAC
8	AseI	ATTAAT
9	TaqI	TCGA
10	NlaIII	CATG
11	MnlI	GAGG/CCTC
12	HpyCh4IV	ACGT
13	DdeI	CTNAG
14	MboII	GAAGG/TCTTC
15	HaeIII	GGCC

Figure S3. Restriction analysis of H1 genome using commercially available restriction endonucleases. Isolated gDNA from the strain H1 was digested (+) with different restriction enzymes. gDNA without enzyme (-) were used as negative control. H1 was resistant to all the tested enzymes as predicted. L: 1 kb ladder. * BccI enzyme was used as control since H1 is sensitive to cleavage by this enzyme. ** The DpnI enzyme cleaves methylated GATC motifs. *** The MboI enzyme cuts unmethylated GATC motifs.
