# Supplementary material for: In Vivo Genome and Methylome Adaptation of cag-Negative Helicobacter pylori during Experimental Human Infection
Source: mBio. 2020 Aug 25;11(4):e01803-20. doi: 10.1128/mBio.01803-20 (PMC7448279; doi:10.1128/mBio.01803-20)
Supplement: FIG S4 [file mBio.01803-20-sf004.pdf]

A)

Strain	H1-H16	8A3	8C10	12A3	12C8	29A2	29C8	48A2	48C8	78A3	78C8	81A1	81C9	87A3	87C7	103A4	103C8	119A2	119C10	125A3	125C7
**G-tract**	9	9	9	13	13	9	8	9	9	9	9	13	13	9	12	9	9	9	9	9	9

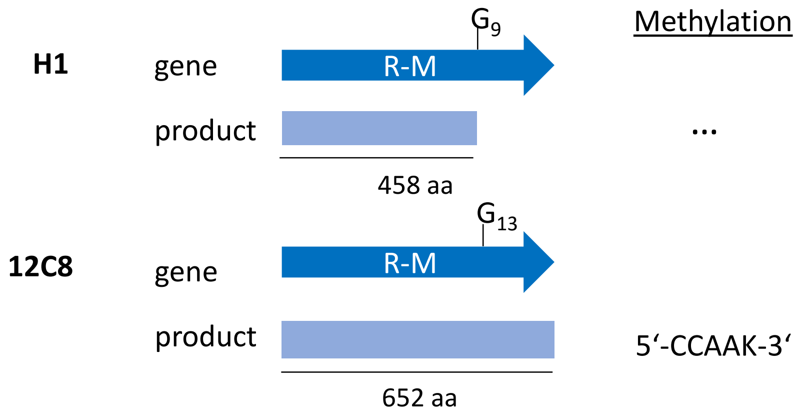

B)

Strain	H1-H16	8A3	8C10	12A3	12C8	29A2	29C8	48A2	48C8	78A3	78C8	81A1	81C9	87A3	87C7	103A4	103C8	119A2	119C10	125A3	125C7
**G-tract**	12	12	12	13	13	12	12	12	13	12	12	13	13	12	13	12	12	12	12	12	12

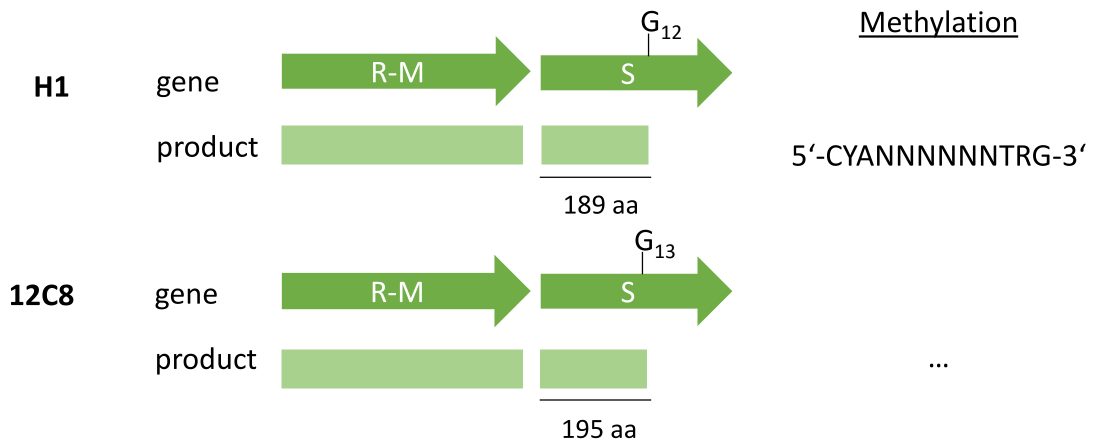

Figure S4. Number of nucleotides within the homopolymeric tracts of the phase-variable R-M systems and graphical representation. (a) R-M system methylating the motif CCAAK. The reisolates with 13 Gs within the homopolymeric tract (highlighted in blue) have an active R-M system. (b) Graphical representation of the R-M system and the S subunit methylating CYANNNNNNTRG. The reisolates with 13 Gs within the homopolymeric tract do not have an active R-M system and therefore, the CYAN$_6$TRG motif is not methylated in those reisolates (green in the panel).
