# Supplementary material for: In Vivo Genome and Methylome Adaptation of cag-Negative Helicobacter pylori during Experimental Human Infection
Source: mBio. 2020 Aug 25;11(4):e01803-20. doi: 10.1128/mBio.01803-20 (PMC7448279; doi:10.1128/mBio.01803-20)
Supplement: TABLE S1 [file mBio.01803-20-st001.pdf]

Table S1. List of *H. pylori* reisolates used in this study and histology scores at the time of *H. pylori* culture from challenged human volunteers (table modified from (Aebischer, Bumann et al. 2008)).

Isolates		1st Gastroscopy (before challenge)		2nd Gastroscopy (6 wpc[a])		3rd Gastroscopy (10 wpc[a])	
**Antrum**	**Corpus**	**Antrum**	**Corpus**	**Antrum**	**Corpus**	**Antrum**	**Corpus**
12A3	12C8	2	2	7	4	7	3
48A2	48C8	0	0	4	5	9	5
78A3	78C8	1	1	7	3	7	3
87A3	87C7	2	1	7	3	5	3
119A2	119C10	0	0	4	2	8	2
8A3	8C10	2	1	4	1	6	4
29A2	29C8	2	2	8	8	10	7
81A1	81C9	-	-	8	3	5	3
103A4	103C8	2	1	12	11	3	2
125A3	125C7	0	0	7	10	7	6

[a]wpc: weeks post challenge

Reisolates from human volunteers given a Ty21a (pUreA/B) vaccine candidate are shown in green and reisolates from human volunteers given the control are highlighted in yellow.
