# Supplementary material for: In Vivo Genome and Methylome Adaptation of cag-Negative Helicobacter pylori during Experimental Human Infection
Source: mBio. 2020 Aug 25;11(4):e01803-20. doi: 10.1128/mBio.01803-20 (PMC7448279; doi:10.1128/mBio.01803-20)
Supplement: TABLE S2 [file mBio.01803-20-st002.pdf]

Table S2. Oligonucleotides (Table S2A) and recombinant bacterial strains (Table S2B) used in this study.

**Table S2A**			
**Primer**	**5' → 3' sequence**	**RS[a]**	**Application**
**Methylome study**			
H1_RM-2_PstI_for	atactgcagATGTTTGAAATGGCAACCG	PstI	Insertion mutagenesis of the target putative R-M system (not assigned to any motif)
H1_RM-2_OL1_rev	cggatcggacagtgcATTCCAGCAATAAGTTGTCT	-	
H1_RM-2_OL2_for	gctcggtagccttgcACTCGAAGCCCTAATTTCAA	-	
H1_RM-2_BamHI_rev	ataggatccATGTATCTTCGTTAATATCGC	BamHI	
H1_MTase3_PstI_for	atactgcagTGCTAAAAGAATATTTAGAAAG	PstI	Insertion mutagenesis of the target putative MTase (not assigned to any motif)
H1_MTase3_OL1_rev	cggatcggacagtgcTCTTTAGCGTTAGTGATAGG	-	
H1_MTase3_OL2_for	gctcggtagccttgcAGCCCAAAACGATTTGAAT	-	
H1_MTase3_BamHI_rev	ataggatccATTGGGTGTTTTAGGGTTTG	BamHI	
H1_RM-4_PstI_for	atactgcagATGGGTGGGCTTTGTTATAAT	PstI	Insertion mutagenesis of the target R-M system (M.HpyH1XX and HpyH1XXP)
H1_RM-4_OL1_rev	cggatcggacagtgcATCCTTATAATTCGTTTCGCT	-	
H1_RM-4_OL2_for	gctcggtagccttgcACACAAACAAAACAAAACCT	-	
H1_RM-4_BamHI_rev	ataggatccTCCTATAATCATCCGGCACT	BamHI	
H1_RM-1_PstI_for	atactgcagATGCAAAATAAAGAAATTTGTG	PstI	Insertion mutagenesis of the target R-M system (M.HpyH1I and HpyH1IP)
H1_RM-1_OL1_rev	cggatcggacagtgcATTCTTGATATAATCCAGCT	-	
H1_RM-1_OL2_for	gctcggtagccttgcACGACGATTAAAAACAGAA	-	
H1_RM-1_BamHI_rev	ataggatccATTAATCAACGCGCTTAATT	BamHI	
H1_RM-1_seq_for	AGTTTTATGCTGGCGGATTG	-	Sequencing of the G homopolymeric tract in M.HpyH1I
H1_RM-1_seq_rev	AAGCAAGTTATAGGGGTGGT	-	
H1_RM-5_For/PstI	atactgcagAACAGCTTATTAGAAAACGCA	PstI	Insertion mutagenesis of the target R-M system (HpyH1II)
H1_RM-5_rev/OL1	cggatcggacagtgcTCCTCTAAATCGCTGTAGAAT	-	
H1_RM-5_for/OL2	gctcggtagccttgcTATTGCAACACGATCCGAT	-	
H1_RM-5_rev/BamHI	ataggatccTGAGGTGGTAGAGCTGATA	BamHI	
H1_S6_PstI_for	atactgcagAACTCTACAAGACTACGCAA	PstI	Insertion mutagenesis of the target putative S subunit (not assigned to any motif)
H1_S6_Ol1_rev	cggatcggacagtgcAGAACCTTGACTGCCTGAA	-	
H1_S6_OL2_for	gctcggtagccttgcACAACCACAAAATCAATGAG	-	
H1_S6_BamHI_rev	ataggatccAGGTCTTATTACCATCGCAT	BamHI	
H1_S7_PstI_For	atactgcagATGGACGCATTAATGCTGC	PstI	Insertion mutagenesis of the target putative S subunit (not assigned to any motif)
H1_S7_OL1_rev	cggatcggacagtgcATGACTGCCCTACCAATAGT	-	
H1_S7_OL2_for	gctcggtagccttgcATGTGAAATGGAATACAGAGCA	-	
H1_S7_BamHI_rev	ataggatccAGCGCTCATTAAATCGTGGT	BamHI	
H1_HP1471_seq_for	ACATGCTAGGGTATTTTCTC	-	Sequencing of the G homopolymeric tract in the target S subunit BcgI like protein
H1_HP1471_seq_rev	AGATTAACTCGTTCTCTACT	-	
H1_HP1471/for	ATTTGTCATCACTGCAGG	-	
H1_HP1471/rev	TAGCTTGTATTTTAGCGC	-	
**Genome study**			
H1_HP1560_seq_for	AACTTACACCACGGTGGT	-	Sequencing of the homopolymeric tract within the target gene
H1_HP1560_seq_rev	ATTATGCATGGCATTGCTTG	-	
H1_HP1560/for	ATGACTACAGACAAAAGCCT	-	
H1_HP1560/rev	AAGCGGTTAGCGATCCTA	-	
H1_hp0164_seq_for	ACGAATTTGACAATTGCATC	-	Sequencing of the homopolymeric tract within the target gene
H1_hp0164_seq_rev	ATCCCCTTCATTCCGCTTA	-	
H1_hp0164/for	AGTTTATTGCCCTTAAGAGA	-	
H1_hp0164/rev	TTTCTTCAACCCCACAAT	-	
H1_HP0770_seq_for	AGAAGAAAAAACCGAACT	-	Sequencing of the homopolymeric tract within the target gene
H1_HP0770_seq_rev	TTAGCCTTAGGGATTTCTT	-	
H1_HP0770/for	ATGGCTGAAGAAGAAAAAACC	-	
H1_HP0770/rev	ATGGGTAGGGTTAGTAACCA	-	
H1_HP0058_for	CTGACTAACAAAAACACTGA	-	Amplification of the target genes
H1_HP0058_rev	TTATGATCCCATATCCACG	-	
H1_jhp0562_for	TTGGATACGGAAAAACTCGT	-	
H1_jhp0562_rev	ACTTTCTGGTGTTTAATTGAT	-	
H1_HP0503_for	TTGATTAACCCTAAACATTT	-	re-sequencing of the homopolymeric C tract of
H1_HP0503_rev	TTAATCAAACTCACAAGGGT	-	

H1_HP0503_seqfor	TATTTAGTGGATTGCGCTTA	-	the target gene (GTAC Mtase gene)
H1_HP0503_seqrev	ATTTATCCACATAATTGGCTA	-	
H1_jhp0562_1_for	ATGTAGCATTAGAGTCTAAT	-	Check deletion in 29C8
H1_jhp0562_2_rev	ACTTGTATCAAATGAACTCCT	-	
H1_jhp0562_3_for	AGAATAAGAAGGTGTATAAAG	-	
H1_jhp0562_4_rev	CTTTATCCAATTCATACTTGATAT	-	
H1_frpB-hp0874 _for	ACAATGAGTTTAAAGTAACTTC	-	Check deletion in 103C8
H1_frpB-hp0874_rev	ATCGCTAGCTATCAATTGAA	-	
H1_HP1472_for1	ATTCGCACAAACTCACTAA	-	Sequencing of the homopolymeric tract within the target gene
H1_HP1472_rev1	ATAAGAAAGGTAATCGGCTAT	-	
H1_HP1472_for2	ATGTTTTCGCTAGCCACCAC	-	
H1_HP1472_rev2	TCAATATAGATCGTTTCTAAAT	-	
H1_upHP0897_for	ATTCCAAATGTCCAATGAATGT	-	Check deletion in 29C8
H1_HP1241_rev	ATCTGAGCACGCTTAAGTCA	-	
jhp0562_1_for	ATTCGTTCAAGAGCTTTATGA	-	Sequencing of the target gene
jhp0562_2_rev	TAACCGGCCGTAGTAGAAAC	-	
jhp0562_seq3_for	AGAGTTATTGCCCCAAGAATTA	-	
jhp0562_seq4_rev	AGGTCTCTTTAATGTTTGCCT	-	
SangSeq_H1_oppA_for	ATGCAAGGGTTTTTCTTCA	-	Sequencing of the target gene
SangSeq_H1_oppA_rev	AAGTGAAACTAAACGGCT	-	
SangSeq_H1_oppB_for	ATATCACTTTGCCCGTTCT	-	Sequencing of the target gene
SangSeq_H1_oppB_rev	TATCAAACTCGCTACCAAA	-	
SangSeq_H1_oppC_for	TATAACGATCCTTATGTGA	-	Sequencing of the target gene
SangSeq_H1_oppC_rev	TAGAAATCACAATGAGTAA	-	
SangSeq_H1_oppD_for	ATCGTTACCCTTATGAATT	-	Sequencing of the target gene
SangSeq_H1_oppD_rev	TTGCAAGTAATAAACGCTA	-	
Check-oppA_For	ATAACGCTCCCATTTTAGC	-	Check 633bp insertion in oppA in H16
Check-oppA_Rev	ACGCATAAATAGAATTGCTA	-	
Check_oppD_For	ACATCGGTATCATTGGCGA	-	Check 57bp insertion in oppD in H4
Check_oppD_Rev	AGGGTAAGCAAAGCGTAAAG	-	
Check-oppA_For2	AGGCTACGCCTTATTTATA	-	Check 633bp insertion in oppA in H16
Check-oppA_Rev2	TAGAGATCCCTGCATAATT	-	
Check_oppD_For2	AGTTTTAAACGCCATGCAG	-	Check 57bp insertion in oppD in H4
Check_oppD_Rev2	TCTAAAGCAGAAGTGGGCTC	-	
qPCR_H1_KatA_for	AGCATTCAAGTGATGCCCGA	-	Measure *katA* expression by qPCR
qPCR_H1_KatA_rev	AACAACATTAGCCGGAGTGA	-	
HPefpF02	GGGCTTGAAAATTGAATTGGGCGG	-	Amplification of the *efp* target gene
HPefpR01	GTATTGACTTTAATGATCTCACCC	-	

[a]RS: Restriction site

Table S2B					
Escherichia coli					
**Strain**	**Code**	**Plasmid**	**Vector**	**Genotype**	**Ab Resistance**
E. coli MC1061	Eco1447	pSUS3407	pUC19 derivative	candidate RM-1 + aphA3 cassette	Amp, Km
E. coli MC1061	Eco1448	pSUS3408	pUC19 derivative	candidate RM-2 + aphA3 cassette	Amp, Km
E. coli MC1061	Eco1446	pSUS3409	pUC19 derivative	candidate MTase-3 + aphA3 cassette	Amp, Km
E. coli MC1061	Eco1449	pSUS3410	pUC19 derivative	candidate RM-4 + aphA3 cassette	Amp, Km
E. coli MC1061	Eco1519	pSUS3415	pUC19 derivative	candidate RM-5 + aphA3 cassette	Amp, Km
E. coli MC1061	Eco1543	pSUS3417	pUC19 derivative	candidate S6 + aphA3 cassette	Amp, Km
E. coli MC1061	Eco1544	pSUS3418	pUC19 derivative	candidate S7 + aphA3 cassette	Amp, Km
Helicobacter pylori					
**Strain**	**Code**	**Genotype**	**Transformed with plasmic**	**Ab Resistance**	
H. pylori BCS100-H1	Bac725	H1 RM-2::aphA3	pSUS3408	Km	
H. pylori BCS100-H1	Bac726	H1 MTase-3::aphA3	pSUS3409	Km	
H. pylori BCS100-H1	Bac727	H1 RM-4::aphA3	pSUS3410	Km	
H. pylori 12C8	Bac728	12C8 RM-1::aphA3	pSUS3407	Km	
H. pylori BCS100-H1	Bac732	H1 RM-5::aphA3	pSUS3415	Km	
H. pylori BCS100-H1	Bac733	H1 S6::aphA3	pSUS3417	Km	
H. pylori BCS100-H1	Bac734	H1 S7::aphA3	pSUS3418	Km	
