# Supplementary material for: In Vivo Genome and Methylome Adaptation of cag-Negative Helicobacter pylori during Experimental Human Infection
Source: mBio. 2020 Aug 25;11(4):e01803-20. doi: 10.1128/mBio.01803-20 (PMC7448279; doi:10.1128/mBio.01803-20)
Supplement: TABLE S3 [file mBio.01803-20-st003.pdf]

Table S3. SNPs (Table S3A), and CNPs (Table S3B) within the BCS 100 strain (clones H1-H16), and CNPs within the reisolates (Table S3C). The SMRT-sequenced genome of clone H1 was used as a reference for the genome alignments.

Table S3A

#	Position (H1)	Strain	SNP type	Reference (H1)	Modification in clone/reisolate	Gene
1	32823	H2<br>H9<br>119A2	non syn	G	A	copA
2	32899	H12	non syn	C	T	copA
3	197061	H7	syn	A	G	vacA
4	297230	H3<br>8A3<br>8C10<br>29A2<br>29C8<br>125A2<br>125C7	non syn	C	T	multidrug efflux transporter
5	714504	H2<br>H3<br>H4<br>H5<br>H6<br>H7<br>H8<br>H9<br>H10<br>H11<br>H13<br>H14<br>H15<br>H16	non syn	G	T	dnaX
6	786947	H11	non syn	T	G	Type I RM system, S subunit II
7	786955	H5	non syn	T	G	Type I RM system, S subunit II
8	786967	H15	non syn	T	C	Type I RM system, S subunit II
9	841716	H13	non syn	A	C	Type I RM system, S subunit II
10	841813	H5	non syn	A	C	Type I RM system, S subunit II
11	896412	H13	non syn	C	T	oppB
12	1211526	H9	syn	G	A	oppC
13	1211970	H5	non syn	G	A	oppC
14	1424010	H7<br>H11	syn	T	G	adenine specific DNA methyltransferase
15	1487697	H3<br>8A3<br>8C10<br>29A2<br>29C8<br>48A2<br>48C8<br>125A2<br>125C7	non syn	G	A	pdxJ
16	1489146	H5<br>H6<br>H10<br>H13<br>H14	non syn	A	G	wecA

Table S3B

#	Position in H1		Strain	CNP	Length (bp)	Gene
	Start	End				
1	841827	841879	H16	3 SNPs (2 non syn + 1 syn)	53	Type I RM system, S subunit
2	841812	841817	H11	2 SNPs (1 non syn + 1 stop)	5	Type I RM system, S subunit
3	841699	841812	H14	3 SNPs (1 non syn + 1 syn + 1 stop)	114	Type I RM system, S subunit
4	787019	787023	H14	2 SNPs (1 non syn + 1 syn	5	Type I RM system, S subunit

Table S3C

H1 coordinates		Strain	Type of modification	Length (bp)	N° of modifications	Gene	Locus_tag in 26695 (HP) or J99 (JHP)
Start	Stop						
2E+05	231645	12A3, 12C8, 81A1, 81C9	CNP	502	8 SNP	omp28, babA	HP1243
3E+05	304469	12A3, 12C8, 81A1, 81C9, 87C7	CNP	17	4 SNP	omp27, hopQ	HP1177
5E+05	485650	12A3, 12C8, 81A1, 81C9, 87C7	CNP	37	3 SNP	omp, hofC	HP0486
6E+05	607673	29C8	CNP	95	8 SNP	LPS biosynthesis protein	JHP0562
7E+05	710655	12A3, 12C8, 81A1, 81C9, 87C7	CNP	21	3 SNP	-	intergenic
8E+05	841763	12A3, 12C8, 81A1, 81C9, 87C7	CNP	25	6 SNP	type I hsdS	HP0848
9E+05	887376	12A3, 12C8, 81A1, 81C9, 87C7	CNP	223	10 SNP	omp19, babB	HP0896
9E+05	888770	12A3, 12C8, 81A1, 81C9, 87C7	CNP	418	5 SNP + 3 bp deletion	omp19, babB	HP0896

Unique CNP is highlighted in grey
