# Supplementary material for: In Vivo Genome and Methylome Adaptation of cag-Negative Helicobacter pylori during Experimental Human Infection
Source: mBio. 2020 Aug 25;11(4):e01803-20. doi: 10.1128/mBio.01803-20 (PMC7448279; doi:10.1128/mBio.01803-20)
Supplement: TABLE S4 [file mBio.01803-20-st004.pdf]

Table S4. Modifications affecting Opp system components within BCS 100 (clones H1-H16) and reisolates (Table S4A) and opp gene status in 75 diverse *H. pylori* genomes (Table S4B).

**Table S4A**[a]					
oppA					
**Start**	**Stop**	**Strain**	**Description**	**Reference (H1)**	**Modification in isolate**
897283	897283	H16	Insertion	-	633 bp
897439	897445	103A4	Indel	7T	6T
oppB					
**Start**	**Stop**	**Strain**	**Description**	**Reference (H1)**	**Modification in isolate**
896412	896412	H13	SNP (non-syn)	G	A
oppC					
**Start**	**Stop**	**Strain**	**Description**	**Reference (H1)**	**Modification in isolate**
1211526	1211526	H9	SNP (syn)	G	A
1211560	1211567	H11	Indel	8A	7A
1211858	1211864	H2	Indel	7C	8C
1211858	1211864	119C10	Indel	7C	8C
1211885	1211885	H5	SNP (non-syn)	G	A
1211998	1211998	119A2	SNP (non-syn)	G	A
oppD					
**Start**	**Stop**	**Strain**	**Description**	**Reference (H1)**	**Modification in isolate**
1213130	1213135	H2-16, all reisolates	Indel	6A	7A
1213476	1213476	125A2	SNP (non-syn)	G	A
1213495	1213495	H4	Insertion	-	57 bp
1213737	1213737	78A3	Stop	G	A
1213789	1213789	87A3	SNP (non-syn)	G	A
1214054	1214056	H15	Indel	3C	2C

[a]Strain H1 was used as reference genome, Start and Stop positions refer to H1

Table S4B[b]

#	Accession number	Strain ID	*oppA*	*oppB*	*oppC*	*oppD*
1	NC_000915.1	26695	+	+	+	+
2	NC_008086.1	HPAG1	-	+	+	+
3	NC_014256.1	B8	+	+	+	+
4	NZ_CP007606.1	BM013B	+	+	+	+
5	NZ_CP007604.1	BM013A	+	+	+	+
6	NC_017741.1	Shi112	+	+	+	+
7	NC_017367.1	F57	+	+	+	+
8	NC_017379.1	Puno135	+	+	-	+
9	NZ_CP007603.1	J166	+	+	+	+
10	NZ_CP006827.1	oki898	+	+	+	+
11	NC_017362.1	Lithuania75	+	+	+	+
12	NC_022911.1	BM012S	+	+	+	+
13	NC_022886.1	BM012A	+	+	+	+
14	NZ_CP006824.1	oki422	-	+	+	+
15	NZ_AP014712.1	ML3	+	+	+	+
16	NZ_CP006821.1	oki112	+	+	+	+
17	NC_017375.1	83	+	+	+	+
18	NC_017360.1	35A	+	+	-	+
19	NZ_CP011484.1	CC33C	+	+	+	+
20	NZ_CP006820.1	oki102	+	+	+	+
21	NZ_CP007605.1	BM012B	+	+	+	+
22	NC_017359.1	Sat464	-	+	+	+
23	NC_011333.1	G27	-	+	+	+
24	NC_017355.1	v225d	+	+	+	+
25	NC_017926.1	XZ274	-	+	+	+
26	NZ_CP010013.1	Hp238	+	+	+	+
27	NZ_CP011482.1	L7	+	+	+	+
28	NC_017378.1	Puno120	+	+	+	+
29	NC_017368.1	F16	+	+	+	+
30	NC_010698.2	Shi470	+	+	+	+
31	NZ_AP014710.1	ML1	+	+	-	+
32	NC_017382.1	51	+	+	+	+
33	NZ_CP006826.1	oki828	+	+	+	+
34	NC_019560.1	Aklavik117	+	+	+	+
35	NC_017358.1	Cuz20	+	+	+	+
36	NC_021218.3	UM066	+	+	+	+
37	NC_017739.1	Shi417	+	+	+	+
38	NZ_CP011483.1	DU15	+	+	+	+
39	NC_012973.1	B38	+	+	-	+
40	NC_017366.1	F32	+	+	+	+
41	NC_014560.1	SJM180	+	+	+	+
42	NC_017740.1	Shi169	+	+	+	+
43	NC_017063.1	ELS37	+	+	+	+
44	NZ_CP006823.1	oki154	+	+	+	+
45	NC_020509.1	OK310	+	+	+	+
46	NC_020508.1	OK113	+	+	+	+
47	NZ_CP006825.1	oki673	+	+	+	+
48	NZ_CP006822.1	oki128	+	+	+	+
49	NC_017354.1	52	+	+	+	+
50	NC_021882.2	UM298	-	+	-	+
51	NC_021216.3	UM299	-	+	-	+
52	NC_021215.3	UM032	-	+	+	+
53	NC_019563.1	Aklavik86	+	+	+	+
54	NZ_CP011485.1	ausabrJ05	+	+	+	+
55	NC_011498.1	P12	+	+	-	+
56	NC_017365.1	F30	+	+	+	+
57	AP014711.1	ML2	+	+	+	+
58	NC_021217.3	UM037	+	+	+	-
59	NC_017742.1	PeCan18	+	+	+	+

60	NC_017376.1	SNT49	+	+	+	+
61	NZ_CP011487.1	PNG84A	+	+	+	+
62	NZ_CP012905.1	7C	+	+	+	+
63	NZ_AP014523.1	NY40	+	+	+	+
64	NC_017733.1	HUP-B14	+	+	+	+
65	NC_017372.1	India7	-	+	+	+
66	NC_014555.1	PeCan4	-	+	+	+
67	NZ_CP012907.1	29CaP	+	+	+	+
68	NC_017371.1	Gambia94/24	+	+	+	+
69	NC_017361.1	SouthAfrica7	+	+	+	+
70	NC_017357.1	908	+	+	+	+
71	NC_017374.1	2017	+	+	+	+
72	NC_017381.1	2018	+	+	+	-
73	NZ_CP011330.1	J99	+	+	+	+
74	NZ_CP011486.1	K26A1	+	+	+	+
75	NC_022130.1	SouthAfrica20	+	+	+	+

[b]Putatively active genes (+) and truncated (-, red).
