# Supplementary material for: In Vivo Genome and Methylome Adaptation of cag-Negative Helicobacter pylori during Experimental Human Infection
Source: mBio. 2020 Aug 25;11(4):e01803-20. doi: 10.1128/mBio.01803-20 (PMC7448279; doi:10.1128/mBio.01803-20)
Supplement: TABLE S5 [file mBio.01803-20-st005.pdf]

Table S5. Genetic changes in adhesin gene *babA* observed in the reisolates from the different volunteers compared to the H1-H16 clones.

Start	Stop	Strain	Type	Description	Reference (H1)	Modification in isolate
231370	231370	8C10	SNP	* (stop)	c	a
232590	232590	8A3	SNP	non syn	g	a
231005	231005	29A2	SNP	non syn	g	a
232243	233454	29C8	deletion	-	-	1211 bp deletion
232829	232834	103A4	deletion	-	6a	5a
230746	230746	125A2	deletion	gaaaaaacat	-	10 bp
232480	232480	103C8	insertion	ctttt	-	5 bp insert
232605	232605	12A3, 12C8, 81A1, 81C9, 87C7	SNP	non syn	t	c
231144	231645	12A3, 12C8, 81A1, 81C9,	CNP	8 SNP	-	-
