# Supplementary material for: In Vivo Genome and Methylome Adaptation of cag-Negative Helicobacter pylori during Experimental Human Infection
Source: mBio. 2020 Aug 25;11(4):e01803-20. doi: 10.1128/mBio.01803-20 (PMC7448279; doi:10.1128/mBio.01803-20)
Supplement: TABLE S6 [file mBio.01803-20-st006.pdf]

Table S6. MTase genes in strain BCS 100 and reisolates, and their cognate REase and S subunits genes.

Number of R-M systems	Position within H1 genome		Type of enzyme	Specificity[a]	Modified base	Type of R-M system	Assignment	*H. pylori* ortholog enzymes	Comment
1	6944	8898	M	CCA**A**K	m6A	Type III	M.HpyH1I	-	Novel phase variable MTase gene. Motif not found before. Methylated in 12A3, 12C8, 81A1, 81C9
	8901	11816	R				HpyH1IP		
2	11940	15812	RM	GCRC**A**	m6A	Type IIG	HpyH1II	-	Novel MTase gene. Motif not found before
3	62571	64610	RM	CY**A**NNNNNNTRG	m6A	Type IIB	HpyH1III	HpyUM032XIII	Phase variable. Methylation absent in 12A3, 12C8, 48C8, 81A1, 81C9, 87C7
	64661	65229	S1				S1.HpyH1III		
	65236	65836	S2				S2.HpyH1III		
4	219029	217884	M	GTNN**A**C	m6A	Type IIP	M.HpyH1IV	M.Hpy30IX	
	219633	218998	R				HpyH1IVP		
5	264412	265401	M	C**A**TG	m6A	Type IIP	M.HpyH1V	M.Hpy57I	
6	302568	302056	R	TCNG**A**	m6A	Type IIP	HpyH1VIP	HpyF17I	
	303826	302528	M				M.HpyH1VI	M.HpyUM032V	
7	361489	362430	M	G**C**GC	m5C	Type IIP	M.HpyH1VII	M.Hpy99III	
8	399176	398628	R	TCNNG**A**	m6A	Type IIP	HpyH1VIIIP	HpyGI	
	400138	399176	M				M.HpyH1VIII	M.Hpy32VIII	
9	475250	476887	M	ATTA**A**T	m6A	Type IIP	M.HpyH1IX	M.HpyFVII	
	476853	477698	R				HpyH1IXP	Hpy66VIIP	
10	481204	482259	M	A**C**GT	m5C	Type IIP	M.HpyH1X	M.Hpy99XI	
	482252	483005	R				HpyH1XP	Hpy66ORF5135P	REase is frameshifted (H1)
11	506456	507484	M	GT**A**C	m6A	Type IIP	M.HpyH1XI	M.HpyFXII	
	507471	508226	R				HpyH1XIP	Hpy501I	
12	844213	845196	M	GGCC	m5C	Type IIP	M.HpyH1XII	M.Hpy35ORF20746P	
	845193	846048	R				HpyH1XIIP	Hpy35ORF20746P	REase is frameshifted (H1)
13	1143316	1144425	M	**C**TNAG	m6A	Type IIP	M.HpyH1XIII	M.Hpy30X	
	1144436	1145128	R				HpyH1XIIIP	HpyHI	
14	1200899	1200141	M	**C**CGG	m4C	Type IIP	M.HpyH1XIV	M.Hpy66IX	
	1201591	1200896	R				HpyH1XIV	Hpy299IX	
15	1202393	1203547	M	TCG**A**	m6A	Type IIP	M.HpyH1XV	M.HpyFII	
16	1370586	1369753	M	G**A**TC	m6A	Type IIP	M.HpyH1XVI	M.HpyFIII	
	1371413	1370580	R				HpyH1XVIP	HpyAIII	
17	1411490	1412335	M	G**A**GG/CCTC	m6A / m5C	Type IIS	M1.HpyH1XVII	M2.HpyAVI	
	1412332	1413030	M				M2.HpyH1XVII	M1.HpyAVI	
18	1423592	1422513	M	G**A**NTC	m6A	Type IIP	M.HpyH1XVIII	M.HpyAIV	
19	1439225	1437954	R	GAAG**A**/T**C**TTC	m6A /m4C	Type IIS	HpyH1XIXP	HpyAII	
	1440017	1439241	M1				M1.HpyH1XIX	M1.HpyAII	
	1440873	1440010	M2				M2.HpyH1XIX	M2.HpyAII	
20	1441122	1443374	M	GGCAA	m6A	Type III	M.HpyH1XX	-	Novel MTase
	1443384	1446275	R				HpyH1XXP		
21	1532818	1532087	R	TGC**A**	m6A	Type IIP	HpyH1XXIP	HpyCH4V	
	1533707	1532811	M				M.HpyH1XXI	M.HpyFVI	
22	?	?	?	VCGR**A**G	m6A	?	?	-	MTase was not identified

[a] Modifed base is represented in bold, and methylated base on the complementary strand is underlined.

Novel motifs and MTase genes described in this study are highlighted in grey.
